# Cancer Heterogeneity and Cancer Cell Plasticity: Molecular Mechanisms and Precision Therapy

**DOI:** 10.1002/mco2.70812

**Published:** 2026-07-02

**Authors:** Hanwen Hu, Zhixing Hao, Lili Li, Huiying Liu, Chenghui Yang, Zhen Wang

**Affiliations:** ^1^ Department of Breast Surgery Second Affiliated Hospital Zhejiang University School of Medicine Hangzhou China; ^2^ Key Laboratory of Tumor Microenvironment and Immune Therapy of Zhejiang Province Second Affiliated Hospital Zhejiang University School of Medicine Hangzhou China; ^3^ Department of Gastroenteric Surgery Second Affiliated Hospital Zhejiang University School of Medicine Hangzhou China; ^4^ Center For Medical Research and Innovation in Digestive System Tumors Ministry of Education Hangzhou China; ^5^ Department of Oncology Second Affiliated Hospital Zhejiang University School of Medicine Hangzhou China; ^6^ Department of Breast Surgery First Affiliated Hospital of Wenzhou Medical University Wenzhou China

**Keywords:** cancer cell plasticity, precision oncology, therapeutic resistance, tumor heterogeneity, tumor microenvironment

## Abstract

Tumor heterogeneity creates selective pressures and ecological niches that enable cancer cell plasticity; conversely, plasticity continuously generates and reshapes heterogeneity. Together, these processes drive tumor progression, therapeutic resistance, recurrence, and divergent clinical outcomes, thereby limiting the durability of precision oncology. In this Review, we synthesize recent advances in our understanding tumor heterogeneity and cancer cell plasticity across spatial, temporal, and molecular scales, and discuss how the tumor microenvironment regulates plasticity through physical, chemical, and biological cues. We further outline three fundamental challenges for precision therapy, namely, target loss, bypass pathway activation, and adaptive cell‐state transitions, and argue that drug resistance is critically shaped by cancer cell plasticity, which varies widely among patients and contributes to heterogeneous therapeutic efficacy. Building on this framework, we propose a next‐generation precision‐oncology paradigm integrating stratified diagnosis, rational combination intervention, and adaptive monitoring. Finally, we discuss how the integration of single‐cell and spatial multiomics, together with artificial intelligence, could enable the development of “digital twin” tumor models to guide individualized therapeutic decision‐making. Collectively, this review provides an integrated conceptual foundation and practical roadmap for overcoming key bottlenecks in precision oncology driven by tumor heterogeneity and cancer cell plasticity.

## Graphical abstract

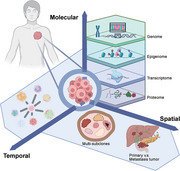

## 1 Introduction

Despite remarkable advances in molecular oncology, durable clinical responses remain the exception rather than the rule for most solid and hematological malignancies. Accumulating evidence indicates that tumor behavior emerges from a highly dynamic system shaped by cellular diversity and adaptive capacity rather than fixed genetic states [1, 2]. Tumor heterogeneity and cancer cell plasticity represent two interdependent dimensions of this dynamic behavior [3]. Heterogeneity provides the substrate—genetic, epigenetic, phenotypic, and spatial diversity—upon which selection acts, whereas plasticity enables cancer cells to reversibly traverse cell states in response to microenvironmental and therapeutic pressures. Importantly, these processes do not operate in parallel but reinforce each other: plastic state transitions generate functional heterogeneity, while pre‐existing heterogeneity biases the trajectories and limits of plastic adaptation [4]. This reciprocal relationship fundamentally challenges conventional precision‐medicine paradigms that assume stable targets and linear evolutionary trajectories [5, 6].

Recent advances in single‐cell, spatial, multiomics, and multimodal technologies have dramatically expanded our ability to depict the panoramic map of tumor heterogeneity and the molecular basis of cell plasticity. However, these technological gains have not been fully translated into conceptual integration. Current frameworks often catalogue heterogeneity across molecular layers without explicitly linking it to the mechanisms governing plasticity, nor do they adequately account for the dynamic alterations of tumor heterogeneity evolution and cancer cell plasticity, resulting in frequent drug resistance and recurrence [7, 8].

**FIGURE 1:**
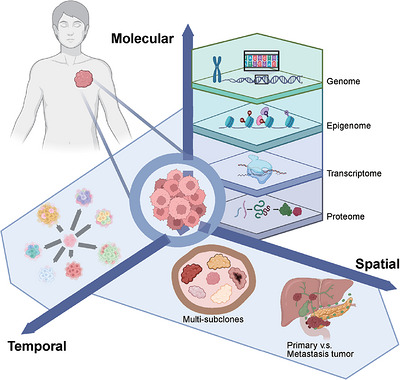
Molecular, spatial, and temporal dimensions of intratumoral heterogeneity. Tumor progression is driven by heterogeneity occurring across multiple biological scales. At the molecular level, tumor cells exhibit alterations across distinct omics layers, including genomic mutations, epigenomic reprogramming, transcriptional changes, and proteomic remodeling, collectively shaping tumor cell phenotypes and functional states. In parallel, spatial heterogeneity arises from the coexistence of multiple genetically and phenotypically distinct subclones distributed across different tumor regions, as well as divergence between primary tumors and metastatic lesions. Moreover, tumor evolution is highly dynamic over time, with sequential clonal expansion, adaptation, and selection generating temporal heterogeneity throughout disease progression and therapeutic intervention. Together, these molecular, spatial, and temporal dimensions establish a complex evolutionary landscape underlying cancer plasticity and treatment resistance.

In this review, we summarize recent advances in understanding tumor heterogeneity and cancer cell plasticity across genetic, epigenetic, transcriptomic, proteomic, and spatial scales. We further propose an integrative, multidimensional framework linking genetic variation, phenotypic switch, and microenvironmental regulation (see Figure 1). With an emphasis on clinical translation, we highlight emerging strategies to monitor and therapeutically modulate heterogeneity and plasticity and discuss their opportunities and outstanding challenges for precision therapy.

## 2 Tumor Heterogeneity

Tumor heterogeneity is not merely a descriptive feature of cancer but a fundamental property that shapes tumor evolution, therapeutic response, and clinical outcome [9, 10, 11]. Across patients, lesions and even neighboring regions within the same tumor, cancer cells, and their microenvironment exhibit extensive diversity in genetic alterations, phenotypic states, and functional behaviors [12]. Clinically, this diversity manifests as striking variability in tumor growth rate, metastatic tendency, and therapeutic response presenting the phenomenon of “distinct treatments for the same disease” and “divergent outcomes under identical therapies” [13]. Crucially, tumor heterogeneity is inherently multidimensional. The spatial dimension determines where we sample; the temporal dimension determines whether the information obtained from sampling at a specific time can represent the future. These dimensions are further layered across molecular hierarchies—from genomic and epigenomic variation to transcriptomic and proteomic diversity—collectively generating a dynamic landscape of tumor states. Importantly, heterogeneity should not be viewed as biological noise, but as the substrate that enables cancer cell plasticity. Pre‐existing diversity biases the range of accessible cell‐state transitions, while plastic adaptation, in turn, actively generates new forms of heterogeneity [14]. In this section, we conduct in‐depth analysis of tumor heterogeneity across spatial, temporal, and hierarchical scales to more comprehensively depict the full picture of tumors, with a particular emphasis on how these dimensions interact with cell plasticity to drive tumor progression, therapeutic resistance, and disease recurrence.

### 2.1 Spatial Heterogeneity

Spatial heterogeneity arises from the nonuniform organization of cancer cells and their microenvironment into distinct structural and functional niches [15, 16, 17]. Driven by spatially patterned clonal lineages, immune infiltration, stromal, and vascular‐neural networks, tumors form ecological mosaics rather than homogeneous cell populations [18, 19]. Even minor differences in microenvironmental composition can impose divergent selective pressures, leading to significantly distinct characteristics of tumor growth dynamics, thereby favoring cancer cell populations with greater survival advantages undertherapeutic exposure [20].

Therefore, spatial heterogeneity fundamentally challenges the prevailing linear strategy of “single biopsies followed by treatment” and motivates the development of multiregional sampling, spatial omics, and regionalized therapeutic interventions.

#### 2.1.1 Intertumor Heterogeneity

Intertumor heterogeneity captures the differences in genes, phenotypes, metabolism, microenvironment, and therapeutic response between different tumor types or different individuals with the same tumor type [21]. These distinctions constitute the biological basis of clinical diversity in disease presentation, therapeutic response, and prognosis. Recognition of this diversity has driven the transition from histopathology‐based diagnosis to molecular classification, fundamentally reshaping modern oncology into personalized and targeted therapies [22, 23].

Oncology treatment paradigms are currently experiencing a fundamental structural transformation, evolving from a framework anchored in anatomical classification and pathological characterization to a precision‐guided management model propelled by molecular subtyping and biomarker stratification. Traditional histopathological classification, although clinically indispensable, has limited capacity to resolve such heterogeneity [24]. In contrast, molecular classification classifies tumors based on the key molecular characteristics of intertumor heterogeneity. Landmark classification frameworks in breast cancer [25, 26], lung cancer, gastric cancer, and urothelial carcinoma [27] have demonstrated that stratifying tumors by driver alterations, transcriptional programs, or immune features can guide effective targeted and immune‐based therapies. These advances established molecular subtyping as a cornerstone of precision medicine and provided a conceptual template for individualized treatment selection across tumor types [28, 29].

However, intertumor classification has improved patient stratification, it remains inherently static. Tumors assigned to the same molecular subtype frequently exhibit divergent therapeutic responses and clinical trajectories, highlighting that subtype identity alone cannot capture adaptive state transitions or evolutionary potential. This classification method is characterized by clear concepts, mature detection, and direct binding to drug therapeutic targets, but it ignores cell lineages to a certain extent.

Recent studies have expanded intertumor classification beyond molecular features to incorporate cell lineage identity, immune architecture, and spatial tissue organization. Studies on spinal ependymomas (such as myxopapillary ependymoma [MPE] and spinal ependymoma [SP‐EPN]) have shown that intertumor heterogeneity is reflected not only in molecular characteristics but also in differences in cell types: MPE tumors are rich in astrocytes, while SP‐EPN mostly presents a ciliated state [30, 31]. These findings suggest that tumors of related lineage may follow divergent differentiation trajectories during development and lineage specification [32]. This classification method emphasizes the decisive role of cell lineages in tumor development. The establishment of cell line atlases also provides a new research platform for other tumor types. Single‐cell analysis by Zhang et al. revealed six colorectal cancer (CRC) immunotypes (G1–G6) characterized by unique TME compositions and evasion mechanisms [33]. These observations underscore the critical importance of cell lineage‐driven classification strategies in unraveling the multifaceted nature of intertumor heterogeneity, thereby offering novel insights into tumorigenesis and lineage specification.

The advent of the spatial omics has reframed tumor classification from cell‐type‐centered schemes to a more complex and comprehensive spatial tissue structure classification. Spatially informed analyses have demonstrated that functional outcomes are determined not only by cellular identity but also by the localization, interaction, and structural arrangement of tumor and immune cells. Three seminal 2020 *Nature* studies established the pivotal role of B cells and tertiary lymphoid structures (TLSs) in immunotherapy [34, 35, 36, 37], shifting the focus from cell‐type identity to spatial‐functional architecture within the “tumor ecological niche.” With the in‐depth study of TLSs, the classification system of TLS has evolved from purely morphological descriptions—such as aggregates and follicles—to a trajectory‐based framework [38]. Notably, a 2025 *Cancer Cell* study classified TLSs into three types (mature, maturing, and divergent) based on TLS developmental trajectories [39]. Beyond TLSs, spatial classification frameworks in glioblastoma (GBM) and small cell lung cancer (SCLC) have revealed that tumor ecological niches and the degree of spatial organization correlate with patient survival and therapeutic sensitivity [40]. In GBM, the “GBmap” single‐cell reference map introduced the tumor structure score, a novel metric that quantifies spatial organization to predict patient survival [41]. This discovery marks the evolution of tumor classification from simple cell type identification to dynamic assessment of spatial structure, further deepening the understanding of the complexity of the tumor microenvironment (TME). Similarly, spatial analysis of SCLC has defined “immune colony niches”—cluster of antitumor macrophages, CD8^+^ T cells, and natural killer (NK) T cells—as predictive biomarkers for immunotherapy response [42]. These two studies not only promote the proposal of “spatial niche” as a new generation of tumor classification dimensions but also provide new ideas for precision medicine. In addition to relatively macroscopic spatial structures, recent studies have also discovered a special three‐cell complex, namely, CD4^+^ T cells and CD8^+^ T cells cobinding to the surface of the same antigen‐presenting cell (APC), which can significantly enhance the antitumor effect [43]. Studies in SCCE and pituitary neuroendocrine tumors further underscore the critical role of the immune microenvironment in tumor progression. Single‐cell analyses revealed the immunosuppressive state, dysregulation of immune cells, and transcription factor regulation within TME is closely related to tumor progression [29, 44]. Collectively, these findings emphasize that intertumor heterogeneity extends beyond intracellular genetic or proteomic variation with tumor–immune interactions, microenvironmental composition, and spatial tissue organization can exert decisive effects on prognosis and therapeutic response. With the advancement of these studies, future cancer treatment will increasingly rely on the comprehensive analysis of tumor spatial organization and microenvironment and achieve more personalized treatment strategies through more refined spatial classification.

Beyond genetic, lineage, and immune‐based stratification, intertumor heterogeneity is increasingly understood as a divergence in functional signaling states, which are often more directly linked to therapeutic vulnerability than genomic alterations alone. Accordingly, tumor classification has gradually expanded from simple gene and transcriptome centric analysis to proteomics and phosphoproteomics, particularly in contexts where therapeutic response and prognosis are tightly linked to pathway activation states. Functional classification based on proteomic and phosphoproteomic analyses have revealed subtypes related to therapeutic response and malignancy. For example, in muscle‐invasive bladder cancer studies, plastic changes in the proteome are closely related to chemosensitivity, and tumors with high pretreatment heterogeneity have a poor prognosis [45]. The phosphoproteomic subtypes of hepatocellular carcinoma (HCC) show that subtype C is associated with increasing malignancy, and potential therapeutic targets have been identified through kinase activity profiles [46]. Thus, multiomics research methods have further targeted tumor classification to therapeutic targets, providing a theoretical basis for clinical treatment.

In summary, intertumor heterogeneity extends beyond genetic and phenotypic differences to encompass spatial structure and functional state diversity (see Table 1). Integrating molecular, spatial, and signaling‐level heterogeneity offers a conceptual and mechanistic foundation for personalized therapy and targeted intervention, while also delineating the inherent limits of static classification systems in clinical decision‐making.

**TABLE 1:** Tumor classification, core heterogeneity features, and heterogeneity‐informed therapeutic strategies.

Tumor type	Classification framework	Representative subtypes	Core molecular programs	Dominant heterogeneity features	Key actionable markers	Heterogeneity‐informed therapeutic strategies	References
Breast cancer	IHC (ER/PR/HER2/Ki67); gene‐expression profiling	Luminal A/B; HER2‐enriched; basal‐like (TNBC)	Hormone receptor signaling; HER2‐driven RTK signaling; EMT and immune‐associated programs in TNBC	Intersubtype divergence in hormone dependence, proliferation and immune exclusion; TNBC exhibits marked spatial immune suppression	ER, PR, HER2, Ki67, CXCL13	Subtype‐matched standard therapy (endocrine or anti‐HER2); mechanism‐based escalation (CDK4/6 inhibition in proliferative luminal tumors); immune‐context‐guided combination in TNBC (chemotherapy ± immunotherapy based on immune architecture)	[47, 48]
Lung cancer	Driver mutations; transcriptomic subtypes; immune phenotypes	EGFR/ALK/KRAS‐driven NSCLC; SCLC‐A/N/P/Y; immune‐hot/cold	Oncogene addiction; transcription factor‐driven lineage states in SCLC; CNV–metabolism–immunity coupling	Profound interlineage heterogeneity (NSCLC vs. SCLC); intralineage state plasticity linked to immune niches	EGFR, ALK, KRAS, SLFN11, DLL3, MT2 niche	Driver‐matched targeted therapy; lineage‐informed chemotherapy in SCLC; immune‐ecotype‐stratified immunotherapy, with DLL3‐ or checkpoint‐based combinations explored according to SCLC state	[42, 49, 50]
Gastric cancer	TCGA molecular classes; transcriptomic and metastatic patterns	EBV^+^, MSI, CIN, GS; ovary‐metastatic subtypes	Immune‐activated EBV programs; hypermutation; RTK amplification; adhesion/differentiation defects	Etiology‐driven immune divergence; metastatic evolution patterns dictate drug sensitivity	EBV, MSI, HER2, CLDN18.2	Etiology‐guided immunotherapy (MSI/EBV); RTK‐targeted therapy in CIN tumors; evolution‐informed treatment adaptation for metastatic subtypes	[51, 52]
Hepatocellular carcinoma	Phosphoproteomics; gross morphology; proteomic subtypes	AFM, PPR, IME, NEU; infiltrative vs. noninfiltrative	Kinase activation states; TGFβ‐driven invasion; immune‐metabolic coupling	Spatially organized immune‐hot vs. immune‐excluded tumors; phospho‐signaling defines aggressiveness	ASPH, TGFβ axis, DPYD, TYMP	Immune‐context‐matched ICI strategies; kinase‐activity‐guided TKI selection; TGFβ/immune cotargeting to overcome infiltrative immune exclusion	[46, 53]
Cholangiocarcinoma	Multiomics + CRISPR dependency; lineage identity; single‐cell states	ICC vs. ECC; biliary vs. squamous lineage; SPINK1^high/low^	Lineage‐specific transcriptional dependency; immune‐recruiting secretory programs	Anatomical and lineage‐dependent vulnerabilities; immune myeloid‐enriched niches in SPINK1^high^ tumors	FGFR2, EGFR, SPINK1	Dependency‐matched targeted therapy (FGFR/EGFR); chemokine–myeloid axis disruption as an adjunct to immunotherapy	[28, 54]
Glioblastoma	Single‐cell + spatial transcriptomics; tumor structure score (TSS)	Metabolic–neural; inflammatory; hypoxic layers	Hypoxia‐driven long‐range regulation; metabolic rewiring; immune exclusion	Highly ordered spatial architecture; hypoxic niches correlate with poor outcome	VEGF, GBP1, DPYD	Structure‐informed therapeutic stratification; antiangiogenic or metabolic modulation to disrupt hypoxic niches; spatially guided immunotherapy exploration	[28, 41]
Small‐cell lung cancer	Transcriptomic states; spatial immune niches	SCLC‐A/N/P/Y; MT2 immune niche	Transcription‐factor‐locked lineage states; DNA damage response heterogeneity	Immune‐colony niches determine prognosis and immunotherapy responsiveness	SLFN11, DLL3, LAG‐3	State‐informed chemotherapy; DLL3‐targeted strategies; immune‐niche‐guided immunomodulation	[42, 55]
Urothelial carcinoma	WES + RNA‐seq consensus classes	Luminal; basal; neuroendocrine‐like	APOBEC mutagenesis; differentiation plasticity	Metastatic lesions show transcriptional reprogramming without major new drivers	FGFR3, NECTIN4	Targeted therapy by dependency; ADC deployment independent of subtype; ICI guided by immune context	[27]
Colorectal cancer	CMS classification; single‐cell states; immune context	CMS1–4; immune‐hot/cold	Stromal‐driven EMT; NETosis‐associated inflammation	Microenvironmental rather than tumor‐cell genetics dominates progression risk	TNFSF14, PPIF	Immune‐guided therapy in CMS1; stroma‐ and inflammation‐modulating strategies combined with standard regimens in CMS4	[33, 56]
Pancreatic ductal adenocarcinoma	Transcriptomic states; metabolic and spatial niches	Classical; basal‐like; necrotic‐front niche	EMT‐linked basal programs; stress‐adapted metabolic states	Necrotic‐front ecological niches drive chemoresistance and plasticity	SPP1, GREM1	State‐aware chemotherapy selection; matrix/CAF reprogramming to relieve immune and drug exclusion; state‐interception strategies [57, 58] rather than cytotoxic escalation	[57, 58]
Ovarian cancer (HGSC)	Structural variation; HRD/HRP; immune context	HRD; HRP subtypes; CD127^+^CD8^+^ T cell‐enriched	DNA repair dependency; replication stress	Immune persistence rather than infiltration predicts outcome	PARP axis, CHK1, CD127	HRD‐guided PARP inhibition; replication‐stress targeting in HRP tumors; immune‐persistence‐informed combinations	[59, 60]
Lymphoma (DLBCL/NKTCL)	Single‐cell ecosystem (LymphoMAPs); viral association	FMAC, LN, TEX; EBV^+^	Immune exhaustion programs; viral‐driven immune suppression	CAR‐T responsiveness dictated by ecosystem state	CD19, BTK, LMP1	Ecosystem‐guided CAR‐T selection; immune exhaustion alleviation; virus‐associated immune reprogramming	[61, 62]
Melanoma	Single‐cell states; anatomical origin; immune phenotype	MM; AM; ALM; antigen‐presenting vs. invasive states	EMT and immune suppression in acral/mucosal tumors	TIGIT^+^ Treg and myeloid dominance in immune‐cold niches	TIGIT	Immune‐checkpoint backbone; immune‐axis‐specific combination in immune‐cold subtypes; state‐restriction strategies	[63, 64]
Prostate cancer	Transcriptomic subtypes; single‐cell lineage states; metastatic site heterogeneity	Luminal‐like; basal‐like; SOX9^+^ AR^low^ stem‐like	Androgen‐receptor signaling; lineage plasticity; stromal‐immune coupling	AR‐dependent and AR‐independent states coexist; basal‐like and stem‐like programs drive therapy resistance and metastatic progression	AR, SOX9, FAP, CXCR6	Lineage‐state‐matched AR targeting; plasticity‐aware combination strategies (AR blockade plus lineage‐constraint or stromal modulation); CAF–Treg niche disruption to prevent immune‐protected state transitions	[65, 66]
Esophageal cancer	Transcriptomic subtypes; single‐cell ecotypes; spatial niche organization	Differentiated; metabolic; immune; stem‐like; EC1–EC5	Differentiation control; epigenetic dysregulation; CAF‐driven spatial niches	Stem‐like and metabolic subtypes show poor prognosis; POSTN^+^ CAF niches impose immune exclusion and therapy resistance	EP300, POSTN, COL17A1, TP63	Subtype‐matched chemoradiotherapy; epigenetic‐state targeting in stem‐like tumors; CAF‐niche disruption to restore immune accessibility	[29, 67, 68]
Thyroid cancer	Differentiation status; single‐cell phenotypes; spatial transcriptomics	Differentiated PTC; dedifferentiated ATC; BRAF‐like; fusion‐driven	MAPK signaling; dedifferentiation; CAF‐vascular coupling	Loss of differentiation correlates with immune suppression and angiogenic remodeling; spatial CAF heterogeneity shapes progression	BRAF, FAP, LAMP5, VEGF axis	Differentiation‐state‐guided MAPK inhibition; immune‐vascular cotargeting in dedifferentiated tumors; CAF‐directed microenvironmental remodeling	[69, 70]
Acute myeloid leukemia	Proteomic subtypes; single‐cell lineage bias; ageing‐associated scores	S1–S8 proteomic classes; stem‐biased vs. differentiated states	Transcription‐factor mutations; stemness maintenance; ageing‐linked programs	Lineage skewing and stem‐like persistence drive relapse risk; ageing‐associated proteomic states predict poor outcome	CEBPA, NPM1, PML‐RARA, ageing score	Subtype‐matched differentiation or cytotoxic therapy; stem‐state‐targeted combinations; age‐adjusted treatment stratification	[71, 72]

#### 2.1.2 Intratumor Heterogeneity

**FIGURE 2:**
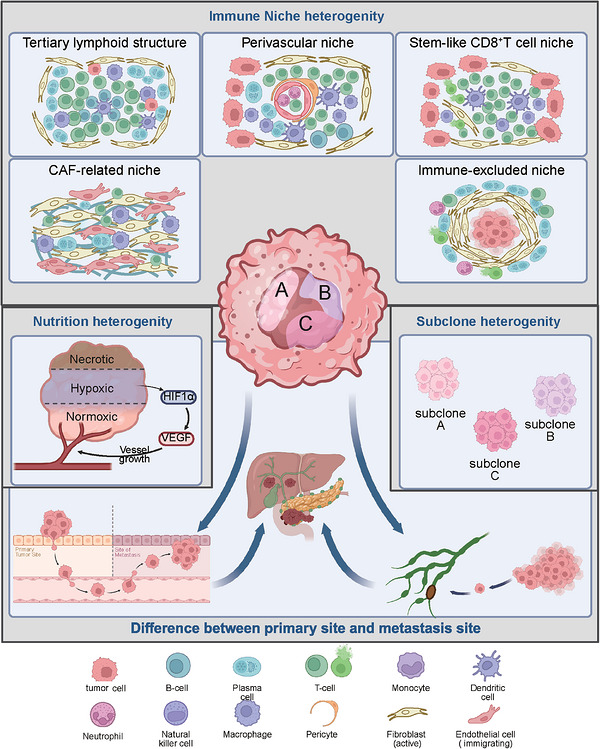
Immune niche architecture and microenvironment‐driven tumor heterogeneity. The tumor immune microenvironment is structured into spatially defined immune niches that contribute to functional heterogeneity and therapeutic outcomes. Distinct immune architectures include tertiary lymphoid structures enriched in adaptive immune cells, perivascular niches supporting immune infiltration, stem‐like CD8^+^ T cell niches sustaining antitumor immunity, CAF‐associated stromal niches promoting immune suppression, and immune‐excluded niches where tumor cells evade immune surveillance. In parallel, microenvironmental gradients in oxygen and nutrients generate compartmentalized regions of normoxia, hypoxia, and necrosis, activating stress–response pathways such as HIF‐1α and proangiogenic signaling (e.g., VEGF). These immune and metabolic pressures collectively shape subclone diversification and selection. Differences between primary tumor sites and metastatic lesions further highlight how immune contexture and microenvironmental remodeling evolve during dissemination, reinforcing spatial heterogeneity across disease stages.

Intratumor heterogeneity (ITH) refers to significant differences in genetics, epigenetics, function, and spatial structure between cancer cell subclones and TME components within a single tumor [73, 74] (see Figure 2). This heterogeneity is usually closely related to acquired therapeutic resistance and recurrence of tumors. Unlike intertumoral diversity, ITH is inherently spatial and dynamic, reflecting ongoing evolutionary processes operating at subregional scales.

Spatial structural heterogeneity represents one of the most intuitive manifestation of ITH, while the existence of spatial subclones is direct evidence of ITH and the basic unit of tumor evolution [75]. Mouse subcutaneous xenograft models derived from single cancer cell lines, illustrate how genetic and epigenetic diversification gives rise to regionally segregated subclones with divergent invasive and proliferative capacities. Specifically, highly invasive subclones are predominantly enriched in the peripheral region of the tumor, while subclones in the tumor center highly express proliferation‐related genes [76]. Correspondingly, the tumor edge typically exhibits a locally “immune‐hot” phenotype driven by immune infiltration, while the tumor core displays features of an “immune‐cold” microenvironment [77]. Such nonrandom topological organization underlies functional compartmentalization and drives tumor adaptation, evolution, invasion, and therapeutic resistance. Based on differences in their cellular components, numerous different terms have been used for description (see Table 2).

**TABLE 2:** Representative spatial structural concepts in the tumor microenvironment.

Spatial structural concept	Definition	References
Immunity hub	A spatially confined region within the tumor microenvironment enriched with immune cells, characterized by intensified immune–immune and immune–tumor interactions, and commonly associated with active antitumor immune responses and improved immunotherapy sensitivity	[42]
Tertiary lymphoid structures (TLSs)	Organized lymphoid aggregates within tumors that recapitulate key architectural and functional features of secondary lymphoid organs, enabling local antigen presentation, lymphocyte activation, and adaptive immune responses against tumor cells	[28]
Tumor immunity barrier (TIB)	Spatially defined tumor regions that restrict immune cell infiltration or function, often formed through physical, metabolic, or stromal mechanisms, thereby facilitating immune exclusion and tumor immune evasion	[57]
CRATER (Cancer Region of Antigen presentation and T cell Engagement and Retention)	A specialized tumor region characterized by coordinated interactions among tumor cells, antigen‐presenting cells and T cells, supporting sustained antigen presentation, T cell engagement and local retention, and thereby amplifying antitumor immune activity	[71]
Niche	A functionally specialized microenvironmental compartment within tumors in which cancer cells, immune cells, stromal components, and vascular elements interact to regulate cell survival, expansion, differentiation and immune escape, ultimately shaping tumor progression	[45]
Three‐cell cluster	Spatially confined multicellular assemblies composed of three interacting cell types that function as minimal instructive units within the tumor microenvironment, coordinating immune regulation, metabolic coupling, and cancer cell plasticity	[43]

Recent technological advances have refined the characterization of ITH across multiple levels, whose core can be summarized as the synergistic effect of spatial distribution differences and multiomics molecular heterogeneity [78]. Integrating advanced imaging technologies and spatial omics methods enables systematic revealing significant differences in biological characteristics between different regions within tumors. These approaches hold particular promise for integrating radiological features with molecular heterogeneity to support clinical decision‐making.

#### 2.1.3 Heterogeneity Between Primary and Metastatic Lesions

Metastasis represents the leading cause of cancer‐related mortality, and “primary‐metastatic ITH” has become a core loophole in the failure of clinical therapy. The heterogeneity between metastatic and primary lesions is the result of the “spatial‐temporal” dual deviation from the genome to the microenvironment during tumor progression, directly affecting therapeutic response and prognostic outcomes [79].

##### 2.1.3.1 Clonal Selection and Genomic Divergence During Metastatic Dissemination

Pan‐cancer whole‐genome profiling shows that metastatic tumors exhibit a modest increase in the overall mutation burden but a pronounced enrichment of genomic variations, and the heterogeneity manifesting distinct tissue‐specific signatures [80]. Lung adenocarcinoma and analogous tumor types display moderate genomic divergence between primary and metastatic lesions, whereas breast, prostate, and thyroid carcinomas undergo profound genomic remodeling during late‐stage metastatic progression [81]. Critically, therapeutic exposure imposes stringent evolutionary bottlenecks that further sculpt the clonal architecture of metastases, favoring the selection and expansion of subclones with adaptive genomic and phenotypic traits [79]. Multiregional genomic sequencing shows that primary lesions usually retain multiple parallel branches, while metastatic lesions are often “initiated” by a few or even a single subclone. For example, large‐scale multiomics of 257 primary regions and 176 metastatic regions in 182 HCC cases revealed that primary subclones rich in hypoxic characteristics are more likely to have polyclonal dissemination, while the neoantigen in metastatic lesions is reduced and T cell reactivity is weakened [82]; among them, subclones without *Wnt* mutations gain a high selection advantage in metastasis, accompanied by immunosuppressive B cells mediating terminal exhaustion of CD8^+^ T cells via the HLA‐E:CD94–NKG2A axis [82].

##### 2.1.3.2 Clonal Seeding and Organ‐Specific Metastatic Ecotypes

A similar clonal selection pattern during metastasis has been observed in prostate cancer, another solid tumor with high metastatic potential. Multiregional sequencing of prostate cancer with synchronous lymph node metastasis found that multiple spatially separated subclones coexist in the primary lesion, but only subclones with specific genomic–morphological characteristics (high Gleason grade + PTEN deletion) successfully “seeded” the lymph nodes, suggesting that metastasis is a screening process of “polyclonal primary to oligoclonal metastasis” [83]. However, there are other metastatic modes; integrated analysis of 607 regions in de novo metastatic prostate cancer shows that approximately 30% of cases have “polyclonal metastatic seeding,” leading to inconsistent genotypes between different metastatic lesions. Clinical simulations show that relying on single‐point biopsy is likely to mismatch key driver genes, while multiregional mixed sequencing can significantly improve the accuracy to 92% [84].

Single‐cell resolution studies have further revealed that metastatic lesions not only “reshuffle” at the genomic level but also show organ‐specific remodeling at the transcriptomic, proteomic, and microenvironmental levels, forming a “metastatic ecotype” significantly different from the primary lesion. Among them, the genomic variations and clonal patterns of CRC peritoneal metastases are roughly similar to those of primary lesions, but the transcriptomic characteristics show obvious mesenchymal transformation and fibrotic microenvironment remodeling [85]. It has also been found that the cancer stem cell‐like subpopulation of CRC shows a clear organ metastatic preference: the subpopulation with high DLL4/MAFA expression tends to metastasize to the ovary, while the cholangiocyte‐like phenotype subpopulation specifically metastasizes to the liver [86]. Beyond the intrinsic organotropism of tumor cell subpopulations, the microenvironmental niche of metastatic sites further imposes strong selective pressure, giving rise to organ‐specific immune landscapes that are markedly distinct from both primary lesions and other metastatic locations. Comparison of brain metastases and leptomeningeal metastases (LM) found that brain parenchymal metastases are rich in CXCL9^+^ macrophages, CXCL13^+^CD4^+^ T cells, and B cells, which can form TLSs and are associated with good tyrosine kinase inhibitor (TKI) response; LM show lymphocyte exhaustion and SPP1^+^ macrophage enrichment, and the expression of blood–brain barrier cell transport protein genes is reprogrammed, leading to an immunosuppressive microenvironment [87]. This tissue‐specific immune remodeling is not unique to central nervous system metastases. The proportion of PD‐L1 negative tumor‐associated macrophages (TAMs) in lung adenocarcinoma brain metastases is significantly increased, and tumor‐infiltrating lymphocytes are reduced, presenting an “immune cold” phenotype, which is associated with poor anti‐PD‐1 efficacy [88, 89].

##### 2.1.3.3 Immune and Stromal Remodeling of Metastatic Niches

Immune privilege and stromal reprogramming represent defining hallmarks of metastatic niches, particularly in gastrointestinal malignancies. Rapid autopsy of 55 primary lesions and liver/lung/peritoneal metastases of pancreatic cancer shows that cancer cells in metastases generally convert to a “basal‐like” state and are spatially adjacent to *TGFB1* expressing myofibroblastic cancer‐associated fibroblasts (myCAFs). Concomitantly, plasma cell exclusion mediated by the CXCR4–CXCL12 axis is significantly enhanced in the edge region of metastases, forming an immune‐privileged microenvironment [90]. Comparable immune–stromal crosstalk shapes the metastatic phenotype of breast cancer, albeit with distinct cellular components. Imaging mass cytometry of distant breast cancer metastases shows that the phenotypic composition of tumor cells in metastases is similar to that of primary lesions, but the total number of immune cells is reduced, the proportion of myeloid cells is increased and presents tissue‐specific phenotypes, suggesting that myeloid cells are one of the core immune regulators of breast cancer metastases [91]. Collectively, these multidimensional studies collectively confirm that the heterogeneity between metastatic and primary lesions is the result of the combined action of clonal evolution, organ microenvironmental selection, and therapeutic pressure, and its molecular characteristics and functional phenotypes provide key targets for the precision treatment of metastatic tumors.

In summary, the heterogeneity between metastatic and primary lesions is reflected in three levels: “genomic selection‐epigenetic reprogramming‐immune remodeling,” and is deeply shaped by the organ microenvironment. Systematically depicting metastasis‐specific evolutionary paths will provide a new “spatiotemporal dual‐targeting” strategy for precision therapy.

### 2.2 Temporal Heterogeneity

**FIGURE 3:**
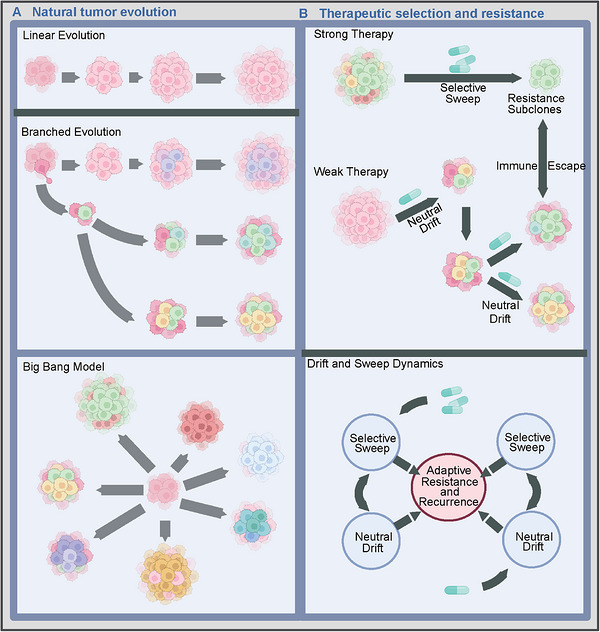
Temporal heterogeneity and evolutionary trajectories of tumors under natural progression and therapeutic pressure. (A) Schematic showing linear, branched, and Big Bang evolutionary models during tumor initiation and progression. Early diversification generates multiple subclones, followed by either selective sweeps or neutral drift depending on tissue context. (B) Timeline depicting selective sweeps under strong therapy versus neutral evolution under weaker pressure, highlighting emergence of resistant subclones, ecDNA amplification, and immune escape pathways. Moreover, conceptual model illustrating repeated transitions between neutral drift and selective sweep across treatment cycles, resulting in stepwise reshaping of clonal architecture and phenotypic states.

While spatial dimensions of ITH have been extensively characterized, temporal heterogeneity emphasizes the continuous, often reversible remodeling of tumor genomes, epigenomes, transcriptomes, proteomes, and immune microenvironments throughout the natural history of cancer—from premalignant lesions through diagnosis, treatment, and eventual recurrence. This dynamic process is driven by the combined effects of clonal evolution and selective pressures imposed by the microenvironment and therapeutic interventions [92, 93]. Temporal heterogeneity not only reveals the evolutionary trajectory of tumors from origin to progression but also directly determines therapeutic response and recurrence risk (see Figure 3), and often evolves synergistically with immune escape mechanisms to jointly shape malignant tumor outcomes, making it a key target for optimizing treatment strategies in the era of precision medicine [94, 95].

#### 2.2.1 Natural Evolution of Tumor Heterogeneity Over Time

In the absence of therapeutic intervention, temporal heterogeneity primarily arises through clonal evolution during natural tumor progression. Three canonical models have been described: linear evolution, in which dominant clones sequentially acquire advantageous mutations; branched evolution, characterized by parallel expansion of multiple subclones; and the Big Bang expansion model, in which extensive early diversification is followed by largely neutral drift [93]. The evolutionary trajectories of different tumors show significant tissue specificity, with distinct phased patterns observed across gastrointestinal malignancies. For instance, the gradual evolution of gastric cancer is accompanied by the occurrence of intestinal metaplasia and mutations in driver genes (such as *ARID1A*, *SOX9*), where different gene mutations correspond to different gastric mucosal niches; once entering the invasive stage, branched evolution occurs, suggesting a phased transition from early neutral expansion to late selective sweep [96]. A comparable stepwise clonal evolution process governs the malignant transformation of esophageal adenocarcinoma, whose precursor lesions (Barrett's esophagus and dysplasia) harbor independent clonal stem cell populations at each stage, and their mutation accumulation trajectories directly point to malignant transformation [97]. Pancreatic intraepithelial neoplasms (PanINs)—the precursor lesions of pancreatic ductal adenocarcinoma (PDAC)—further exemplify the multifocality and clonal independence of premalignant evolution: hundreds of PanINs almost all carry *KRAS* hotspot mutations, yet most originate from independent clones, and some even show polyclonal origin characteristics [98].

During the progression from precancerous to malignant CRC, clonal dynamics cooperate with microenvironmental remodeling; the chromosomally unstable (CIN) subtype accumulates immune exclusion (IEX) characteristics with pseudotime progression, which is associated with poor prognosis [99]. Similar genotype–phenotype coevolution is observed in hematological malignancies: dynamic changes in genomic drivers, transcriptional characteristics, and microenvironmental interactions constitute the core of heterogeneity during the transformation of precursor multiple myeloma to malignancy [100]. In the head and neck squamous cell carcinoma (HNSCC), as lesions progress from normal tissue to metastases, the interaction between LGALS7B^+^ malignant cells and CXCL8^+^ fibroblasts continuously enhances with clonal evolution [101]. Breast cancer represents a notable exception to the typical “late‐stage clonal expansion” paradigm, as clonal expansion initiates as early as the precancerous stage; driver events such as der (1;16) can be acquired in early adolescence, and after decades of evolution, form malignant tumors of polyclonal origin [102]. This “polyclonal ocean” model is echoed in 3D whole‐tumor perspective studies of GBM, which originate from neural developmental lineages, undergo chromothripsis initiation, and intratumor subclones undergo restricted expansion along the “core–edge–contrast enhancement” axis [103]. The inductive role of extrinsic environmental factors in shaping temporal heterogeneity is most fully reflected in lung cancer; during lung adenocarcinoma evolution, 22 of the 40 common cancer genes undergo significant subclonal selection, and the timing of whole‐genome duplication is closely related to recurrence risk. Among them, the evolutionary path of tumors induced by environmental factors such as smoking is inherently different [104]. These researches result collectively confirm that temporal heterogeneity during natural progression is an inevitable result of tumors adapting to the environment and gradually becoming malignant, and its evolutionary trajectory can be accurately depicted through mutation timing, clonal expansion mode, and microenvironmental adaptability.

#### 2.2.2 Therapy‐Driven Temporal Heterogeneity and Adaptive Resistance

With the continuous progress of tumor treatment, therapeutic intervention, as a strong selection pressure, significantly accelerates the accumulation of temporal tumor heterogeneity. Under effective treatment, selective sweeps rapidly eliminate sensitive clones, enabling pre‐existing or newly acquired resistant subclones to dominate, forming a “bottleneck to monoclonal” replacement. This evolutionary pattern under strong selection pressure is very common in clinical practice. For example, SCLC shows clonal homogeneity before first‐line platinum chemotherapy; after first‐line platinum‐based chemotherapy, intratumor genomic heterogeneity and spatial clonal diversity erupt; after sensitive subclones are damaged, cryptic subclones carrying mutations in *TP73*, *CREBBP/EP300*, or *FMN2* re‐expand and are mutually exclusive with *MYC* family amplification, leading to 95% recurrence within 6 months [105, 106]. In *EGFR*‐mutant NSCLC, increases in EGFR extrachromosomal DNA (ecDNA) copy number can be detected in circulating tumor DNA within weeks of osimertinib therapy, signaling rapid emergence of resistant subclones [107]. This evolutionary mode reflects the temporal characteristics of the exhaustion of highly sensitive subclones after potent treatment and the exponential expansion of newly developed drug‐resistant clones.

In contrast, neutral evolution dominates under weaker selection, with multiple subclones coexisting through stochastic drift. In metastatic melanoma, mesenchymal‐like states driven by *TCF4* are already present in early biopsies of immune checkpoint blockade nonresponders; epigenetic targeting of *TCF4* restores antigen presentation and T cell infiltration, demonstrating reversibility during neutral evolution [108]. In HER2‐positive gastric cancer, approximately one out of three of the cases show EMT accompanied by upregulation of PD‐L1 and CCL2 after trastuzumab resistance, but no single dominant driver clone, suggesting multipathway parallel resistance under neutral drift [109]. More complex “drift–sweep–redrift” dynamics can occur across treatment cycle [93], as illustrated in CRPC patients, both AR‐active and neuroendocrine subtypes coexist in multifocal metastases, AR clones are swept under abiraterone pressure, and NE subtypes drift randomly; when switching to PARP inhibitors, *BRCA2*‐mutant clones are swept again, showing “stepwise” temporal heterogeneity [110]. Comparison of pre‐ and posttreatment biopsies of metastatic melanoma found that B2M, JAK1/2 inactivation, and *SEC24C/D* mutations evolve downward through two pathways: disrupting antigen presentation and *STING* transport, reducing Type I interferon production and cytotoxic T cell activation, indicating that immune escape can evolve in parallel through multiple pathways under immunotherapy pressure [111]. This selection mechanism of therapeutic pressure suggests that drug resistance is not dominated by a single clone but by the slow accumulation of multiple subpopulations.

#### 2.2.3 Coevolution of Tumor Cells and the Microenvironment

In addition to cancer cells forming heterogeneity through natural evolution and microenvironmental adaptation in the temporal dimension, immune cells, stromal cells, and vascular systems in the TME also undergo dynamic remodeling with tumor progression. A study covering paracancerous normal tissue (n‐lung), adenocarcinoma in situ, minimally invasive adenocarcinoma, and invasive adenocarcinoma of NSCLC confirmed that as tumors progress from precancerous lesions to invasive stages, the maturity of tumor‐associated TLSs also gradually increases [112]. When small tumor masses (usually less than 1–2 mm) grow, nutrients diffused from surrounding tissues can no longer meet the demand. At this time, cancer cells will release a large amount of “vascular endothelial growth factor (VEGF),” activate and attract endothelial cells of nearby blood vessels; these endothelial cells begin to proliferate and migrate, forming a new, tortuous, and dysfunctional vascular network that penetrates into the tumor interior—a process called “tumor angiogenesis.” Cancer cells can also promote the proliferation and migration of lymphatic endothelial cells by releasing key growth factors such as VEGF‐C and VEGF‐D, thereby promoting lymphangiogenesis, a process called “tumor lymphangiogenesis” [113].

In summary, temporal heterogeneity is driven by the dual wheels of “natural evolution” and “therapeutic selection.” Undeniably, immune escape is a key manifestation of temporal heterogeneity under therapeutic pressure, which is likely to form a positive feedback loop of “immune editing‐escape.” Therefore, real‐time, multiscale, spatially resolved monitoring and intervention will become the next breakthrough to overcome drug resistance and achieve “evolution‐controllable” oncology.

### 2.3 Molecular Heterogeneity

Spatio‐temporal heterogeneity of tumors further extends to intricate molecular variations, namely, molecular heterogeneity, which represents the core intrinsic manifestation of tumor heterogeneity. This feature is not a unidimensional molecular alteration but multilayered molecular disorders across the genome, transcriptome, and proteome. Meanwhile, dynamic regulatory differences in posttranscriptional modifications further amplify molecular diversity, collectively shaping the heterogeneous phenotypes of tumor cells and laying a foundation for subsequent analysis of heterogeneous characteristics and regulatory mechanisms at each molecular level.

#### 2.3.1 Genomic Heterogeneity

Genomic heterogeneity represents the foundational and most extensively studied layer of ITH, encompassing sequence‐level differences among cancer cells within the same tumor, including somatic mutations, copy number variations (CNVs) and chromosomal structural alterations accumulated during rapid proliferation [114].

From an evolutionary perspective, somatic mutations can be broadly classified into truncal and branching mutations based on their clonal distribution. Truncal mutations—also termed founder mutations—are present in all cancer cells and typically arise early during tumor evolution. Acquired by the tumor‐initiating cell, these mutations are propagated to all descendant cells and form the shared genetic backbone of the tumor, occupying the “trunk” of the phylogenetic tree [115]. They often affect key driver genes and confer survival or proliferative advantages essential for tumor initiation. By contrast, branching mutations emerge later and define genetically distinct subclones [115].

The heterogeneity of driver gene mutations is the focus of genomic heterogeneity research because it is directly related to tumorigenesis, development, and response to targeted therapy [116]. Within tumors, the mutation status of driver genes may have significant spatial differences. On the one hand, some core driver gene mutations may exist as “truncal mutations” in all cancer cells and are initiating events of tumorigenesis. For example, in acute myeloid leukemia (AML), *NPM1* serve as a key driver; notably, the *NPM1*‐high expression subpopulation coexists with the *FLT3‐ITD* mutant subpopulation, while stem cell‐like AML subpopulations are more prone to harbor *TP53* mutations [71, 117, 118]. Similarly, in PanINs, nearly all lesions carry oncogenic *KRAS* hotspot mutations, while diverse somatic mutation profiles [98]. In mixed neuroendocrine–non‐neuroendocrine neoplasms, adenocarcinoma and neuroendocrine carcinoma regions share core driver alterations but exhibit compartment‐specific mutational expansion [119]. These examples illustrate that even shared driver events can be differentially expressed or functionally deployed across subclones. On the other hand, mutations in noncritical driver genes may exist only in some subclones as “branching mutations” or coexist with “truncal mutations” in different subpopulations. Study has found that multiple different breakpoints of EGFRvIII can be identified in multiple tumors through the Split Read method, revealing the subclonal origin and ITH of this mutation [120]. Beyond nuclear DNA, somatic mitochondrial DNA mutations also display pronounced heterogeneity, particularly in leukemia, where the coexistence of mutant and wild‐type mtDNA balances mitochondrial dysfunction and cellular adaptability, driving metabolic reprogramming and therapeutic resistance [121]. Such diversity explains why single‐agent targeted therapies frequently fail: under therapeutic pressure, untargeted subclones can be positively selected, ultimately driving relapse.

CNVs represent another major source of genomic heterogeneity by altering gene dosage across large genomic segments. CNVs can modulate the expression levels of numerous genes, thereby perturbing cellular biological behaviors. Notably, different subclones may carry different CNV events, which may be related to tumor invasion, metastasis, or drug resistance. For example, in different region of high‐grade serous ovarian carcinoma, CNVs related to homologous recombination deficiency—including HRD‐DUP (*BRCA1* mutation‐like) and HRD‐Del (BRCA2 mutation‐like)—are mutually exclusive with fold inversion‐driven CNVs; these two CNV patterns correspond to inflammatory and immunosuppressive TMEs, respectively. Additionally, multiregional sequencing of GBM shows that the frequency of chromosomal segment amplification/deletion (such as EGFR amplification, chromosome 13 deletion) differs significantly between the tumor core and edge regions [122]. In response to this genomic heterogeneity, Clonalscope, a subclone detection method using copy number profiles, identifies malignant cells in tumor tissues by detecting CNVs in gastrointestinal tumors and further identifies the corresponding subclone subgroups and related gene expression spatial maps [123].

Collectively, genomic heterogeneity manifests as clonal diversity, referring to the existence of multiple cell clones with different genetic backgrounds within tumors. All descend from one founding cell, yet as the disease unfolds they continually branch off, accruing fresh genetic lesions that endow daughter subclones with distinct biological identities. By profiling the genomes of geographically separated tumor samples, investigators can reconstruct patient‐specific evolutionary trees, tracing the step‐wise divergence from trunk to twig and revealing the intricate labyrinth through which cancer emerges and progresses.

#### 2.3.2 Epigenomic and Chromosomal Heterogeneity

Beyond DNA sequence alterations, ITH is further shaped by epigenomic and chromosomal variability [124, 125]. Epigenomic heterogeneity encompasses variations in DNA methylation, histone modification, chromatin accessibility, and noncoding RNA regulation among tumor subpopulations, without alterations to the underlying DNA sequence. These epigenetic programs directly modulate gene expression, cooperate with genomic alterations to drive tumor progression, and substantially expand phenotypic diversity.

DNA methylation patterns vary markedly across cancer cell subpopulations and exert strong effects on transcriptional output. In NSCLC, DNA methylation acts in concert with CNVs to regulate oncogene expression through dosage‐compensation mechanisms [116]. Similarly, in bladder cancer, *TM4SF1*‐positive cancer cell subpopulations acquire stem cell‐like characteristics via epigenomic reprogramming, driving the generation of transcriptionally heterogeneous progeny lineages [126]. Such epigenomic reprogramming‐driven subpopulation heterogeneity is not unique to solid tumors; in hematological malignancies such as peripheral T‐cell lymphoma (PTCL), epigenomic dysregulation exhibits subtype‐specific features: among the three subtypes of different molecular classifications in PTCL, only the HMA subtype is characterized by dysregulation of histone methylation and acetylation, yet epigenomic targeted therapy has potential therapeutic efficacy across all three subtypes in clinical treatment [127]. Studies have also found that the promoters of some tumor suppressor genes (such as *EPHA7*, *PCDH10*, *DOK1*) are hypermethylated in some tumor regions, which may lead to the inhibition of the expression of corresponding genes in these regions [128].

Histone modification is often linked to chromatin accessibility [129]; the heterogeneity of histone posttranslational modifications (such as H3K4me3, H3K27ac) and chromatin accessibility determines subpopulation‐specific gene expression. Single‐cell ATAC‐seq analysis of GBM shows that the chromatin open regions of cancer cells in the core and edge regions are significantly different, and the binding activities of *AP‐1* and *BACH1* transcription factors are inversely distributed, regulating the switch between invasive and proliferative phenotypes [130]; in medulloblastoma, chromosomal segregation events lead to differences in chromatin accessibility between subclones, affecting the expression and activation of cancer driver genes [131]. These findings emphasize that epigenetic heterogeneity as a key dimension of ITH, which is closely intertwined with genomic variations. Specially, the crosstalk between histone modification/chromatin accessibility and chromosomal dynamics lays the foundation for understanding the regulatory role of the chromosome set in shaping ITH, bridging epigenetic regulation with chromosomal‐level heterogeneity.

Chromosomal heterogeneity, often referred to as chromosomal instability (CIN), represents a macroscopic manifestation of genomic diversity and is a hallmark of many solid tumors [132]. CIN leads to heterogeneity in chromosome number (such as polyploidy, aneuploidy) and structure (such as translocation, deletion) between cancer cell subtypes. In NSCLC, highly aneuploid subpopulations show significantly enhanced reactivity to radiotherapy combined with immune checkpoint inhibitors [133]; in pancreatic cancer, mesenchymal lineages highly related to EMT show abnormal chromatin accessibility, leading to mitotic delay and increased chromosomal segregation events, driving their unique chromosomal heterogeneity [125]. In addition to chromosome number and structure, abnormal segregation is also an important cause of differences. Abnormal mitotic chromosome segregation can in turn affect histone posttranslational modifications, leading to obvious positional bias between promoters and distal or intergenic regions, affecting subsequent gene expression and causing genetic abnormalities [129].

In addition to chromosomal DNA, studies have also found that ecDNA also plays an important role in tumor heterogeneity. As an important carrier of oncogene amplification, the copy number of ecDNA varies dynamically in cancer cell subclones and is a key driving factor of ITH. Random segregation of ecDNA is an important source of chromosomal heterogeneity, and ecDNA species encoding different oncogenes can achieve coordinated inheritance through mitotic cosegregation, enhancing subclone‐specific malignant phenotypes [134]. These effects are manifested across diverse tumor types via distinct mechanisms, including structural remodeling of ecDNA, adaptive responses to microenvironmental stress, and promotion of CIN. In the context of genomic structural rewiring, GBM exhibits ecDNA structural variation drives enhancer hijacking or loss, resulting in heterogeneous expression of identical genes across subclones [122]. Beyond structural changes, ecDNA mediates adaptive evolution to microenvironmental stress: in pancreatic cancer, MYC‐containing ecDNA enables reversible adaptation to microenvironmental stress and becomes enriched under chemotherapy [135]. Similarly, in SCLC, MYC‐family ecDNA facilitates transcriptional amplification and drives transitions between neuroendocrine and non‐neuroendocrine states [136]. Finally, ecDNA contributes to chromosomal instability cascades. In medulloblastoma, ecDNA‐positive subclones exhibit increased CIN, with greater structural complexity in recurrent tumors [137]. Together, these findings highlight epigenomic and chromosomal heterogeneity as core engines of intratumor diversity, enabling rapid phenotypic adaptation beyond what can be explained by DNA sequence variation alone.

#### 2.3.3 Transcriptomic Heterogeneity

Transcriptomic heterogeneity refers to differences in gene expression profiles between different cells or subpopulations within tumors. As a direct functional manifestation of genomic and epigenomic heterogeneity, it determines the phenotypic and functional diversity of cells [138]. Importantly, transcriptomic heterogeneity is not restricted to malignant cells but is also pervasive across nonmalignant components of the TME, including immune, stromal, and endothelial cells. Interrogating transcriptomic heterogeneity enables the delineation of cellular subpopulations, reconstruction of developmental trajectories, and identification of molecular programs that drive tumor progression, invasion, metastasis, and drug resistance [139].

Gene expression within tumors is rarely uniform and often displays pronounced spatial patterning. These differences reflect variations in the microenvironment, clonal origin, and functional status of cancer cells in different regions; that is, multiple functionally specialized transcriptional subpopulations exist within the same tumor, such as stem cell‐like, proliferative, invasive, and immune evasive subtypes. This spatial transcriptional heterogeneity is widely observed across diverse tumor types. For example, spatial transcriptomics profiling of SCLC has identified mutually exclusive transcriptional subtypes (e.g., *ASCL1*, *NEUROD1*, and *YAP1*), which are spatially segregated within the tumor [140]. Similarly, in lung adenocarcinoma, a “transitional transcriptional subtype” has been identified at the tumor–normal interface, characterized by three core features: coexpression of the epithelial marker CDH1 and the mesenchymal marker VIM; high expression of chemokine modules CCL20 and CXCL13 that recruit neutrophils to establish an “invasion‐supporting niche”; and upregulation of oxidative phosphorylation (OXPHOS) genes such as *NDUFB8* and *SDHB*, consistent with adaptation to local oxygen tension in adjacent normal tissue [141]. Beyond lung cancers, spatial transcriptomic heterogeneity also exhibits tissue‐specific characteristics that guide clinical practice. In olfactory neuroblastoma, on the basis of three molecular classifications, five unique gene expression patterns of malignant epithelial cells have been identified, further guiding chemotherapy regimens. In breast cancer, there are distinct transcriptional subpopulation distributions between different pathological grades: low‐grade tumors are enriched in immune‐regulatory subpopulations such as CXCR4^+^ fibroblasts and IGKC^+^ myeloid cells, while high‐grade tumors are dominated by invasive subpopulations with activation of the MDK signaling pathway [142]. Additionally, studies on bladder cancer have found that recurrent bladder cancer has higher ITH and more active interaction between cancer cells and fibroblasts [143]. Collectively, these all reflect that even within the same molecular subtype, the intratumor transcriptome changes with spatial distribution and further shapes the tumor immune microenvironment (TIME).

A defining feature of transcriptomic heterogeneity is its dynamic plasticity, whereby cancer cells can transition between transcriptional subtypes to adapt to environmental and therapeutic pressures. This plastic characteristics have been observed in multiple tumor types. In melanoma and lung cancer, cancer/testis antigen (CTA) expression is highly heterogeneous: while some CTAs (e.g., *PRAME*) are relatively uniform, others (e.g., *NY‐ESO‐1*) are subpopulation‐specific, and their expression states can switch under treatment [144]. In GBM, the transcriptome of tumor core cells differs significantly from that of infiltrating cells in the surrounding brain regions, which infiltrating cells are more likely to express oligodendrocyte precursor‐like characteristics [130]. In bladder cancer, *TM4SF1*‐positive cancer subpopulations can not only evolve from ancestral cells with different expression profiles but also show transcriptional plasticity, promoting the development of transcriptionally heterogeneous progeny cell lineages [126]. Notably, this transcriptional subtype switching is not random but a directional process achieved through the switching of master regulators, with clear trigger signals and regulatory networks, including enhancers and noncoding RNAs. For example, in melanoma, BRAF inhibitors can induce the downregulation of melanocyte differentiation transcription factor (*MITF*), thereby activating the AXL–MEK pathway, driving the transcriptional subtype switch from “differentiated” (MITF‐high) to “undifferentiated” (AXL‐high). Importantly, MITF‐high expression phenotype can be restored after drug withdrawal [144]. In lung adenocarcinoma, TGFβ1 secreted by CAFs can induce SMAD2/3 phosphorylation, activate *SNAI1* transcription, and promote the switch from “proliferative” (Ki67‐high) to “invasive” (VIM‐high) subtypes [141]. In acute lymphoblastic leukemia (ALL), the *KMT2A:AFF1* fusion protein can induce the activation of “subtype‐specific enhancers,” which only bind to the *IRF4* gene in the switched subtype, driving the expression of immune escape‐related genes [145]. In esophageal cancer, in the SERPINH1‐high expression subpopulation, lncRNA HOTAIR binds to EZH2 (histone methyltransferase [HMT]) to inhibit the expression of CDH1 (epithelial marker), maintaining the mesenchymal‐like transcriptional phenotype [146]. Thus, transcriptomic heterogeneity is integral to tumor evolution: it captures phenotypic and functional diversity and provides mechanistic insights.

Ultimately, transcriptomic heterogeneity transforms seemingly homogeneous tumors into a functionally organized “microdomain mosaic.” By resolving the transcriptional programs that underlie cancer cell phenotypes, transcriptomic profiling supports prediction of therapeutic response and evolutionary trajectories and can thereby inform clinical decision‐making.

#### 2.3.4 Proteomic Heterogeneity

Proteomic heterogeneity refers to differences in protein expression, modification, and function between distinct cells or regions within the same tumor. As the final product of gene expression, proteins are the main executors of cellular functions; therefore, proteomic heterogeneity directly reflects the functional diversity of cancer cells. This heterogeneity is reflected not only in protein expression levels but also in posttranslational modifications, such as phosphorylation, ubiquitination, subcellular localization, and interaction networks of proteins [147].

The protein expression profiles of various cancer cell subtypes are significantly different, forming functionally specialized proteomic subtypes. For example, proteomic analysis of mantle cell lymphoma has identified three subtypes: high‐risk (*TP53* mutation), intermediate‐risk (*ATM* mutation), and low‐risk (no key gene mutations), corresponding to protein characteristics of enhanced proliferative metabolism, activated angiogenesis, and proinflammatory responses respectively [148]. Proteinomic subtype classification based on functional expression profiles is also applicable to solid tumors. Proteomic analysis of cribriform ductal carcinoma (CDC) shows that ribosomal biosynthesis proteins are the most significant malignant characteristics, and high RPF2 protein expression is associated with poor prognosis [149]. In addition, some cancer‐related proteins can effectively guide the spatial division of tumor edges. In high‐grade glioma, the expression of EGFR protein at the resection edge is 3.8 times higher than that at the core, and it colocalizes with TEAD1, suggesting that TEAD1 directly regulates its transcription [150]. Spatial heterogeneity of proteins is not limited to cancer cells but also extends to immune components in the TME. Myeloid single‐cell proteomics reveals that VEGFα^hi^ neutrophils and SPP1^hi^ macrophages in triple‐negative breast cancer (TNBC) colocalize in the hypoxic region of the tumor core, forming an angiogenic niche [151]. Beyond differences in protein expression levels, posttranslational modifications represent a critical layer of proteomic heterogeneity, further expanding the functional diversity of cancer cells. The intratumor “microregional” differences in protein posttranslational modifications can indicate tumor‐related functional classifications. Combined with imaging mass spectrometry and laser capture microdissection, the phosphorylation levels of ERK, STAT3, and AKT in the same CRC tumor can differ by more than 10 times and are not completely consistent with *KRAS* mutation abundance, suggesting “genome‐proteome” decoupling [152]. Single‐cell proteomics of TNBC found that the UQCRFS1‐high expression subpopulation has enhanced mitochondrial respiratory chain Complex III activity, elevated reactive oxygen species (ROS) levels and activation of the NRF2–PD‐L1 axis, thereby promoting immune escape. Spatial localization shows that this subpopulation is more than 100 µm away from CD8^+^ T cells, forming a “metabolic–immune” dual barrier [153].Collectively, these findings underscore the unique value of spatial proteomics for resolving tumor architecture and functional niches, often outperforming transcriptomics in capturing posttranslational regulation and pathway activation states that more directly reflect downstream oncogenic signaling. This “genome–proteome” decoupling phenomenon indicates that proteomic heterogeneity cannot be fully predicted by genomic characteristics, highlighting the irreplaceable value of direct proteomic profiling in deciphering tumor heterogeneity and guiding precision therapy.

## 3 Cancer Cell Plasticity

“Plasticity” is one of the most daunting traits of malignant tumors, enabling cells within the same tumor can undergo phenotypic switching within hours to days, transitioning from quiescence to proliferation, epithelium to mesenchyme, drug sensitivity to resistance, and immunogenicity to immune evasion [154]. Driving this “shape‐shifting” ability is not only the inherent genomic instability of cancer cells but also the regulatory effects of their surrounding microenvironment (TME). Via the sophisticated regulation of multidimensional physical, chemical, and biological signaling networks, the TME endows cancer cells with the ability to dynamically reconfigure phenotype in response to external pressures, sustaining adaptation and therapeutic escape, and disease progression.

### 3.1 Mechanical Cues of the TME: Stiffness, Viscoelasticity, and Tissue Fluidity

The physical properties of tumor tissue, including matrix stiffness, interstitial fluid pressure [155], mechanical tension, fluid shear stress, and tissue fluidity, are associated with cancer cell cytoskeletal reorganization, extracellular matrix (ECM) composition, and cellular structures within the TME. Stiffness gradients are a shared feature of a broad spectrum of malignancies, such as breast cancer, liver cancer, and PDAC [156]. The mechanical landscape of solid tumors exhibits a heterogeneous distribution characterized by “hard periphery, soft core, and gradient at the margin.” This architecture is well‐illustrated in GBM, where combined application of intraoperative 3D neuronavigation and nanoindentation has shown that the tumor core has a stiffness of approximately 200–400 Pa, while the margin can increase to 1500–2000 Pa, in contrast to normal brain tissue that is only about 100–300 Pa [103]. Tumor cells exploit these gradients through durotaxis—an affinity for stiffer ground akin to pedestrians choosing paved roads. This phenomenon is predominantly governed by actin‐rich protrusions. Upon encountering regions of increased matrix stiffness, pseudopodia sense mechanical cues that promote focal adhesion stabilization, thereby reinforcing further protrusive activity and surrounding microenvironmental probing [157]. Within this mechanotransductive framework, the small GTPase Rac functions as a central molecular transducer that couples extracellular stiffness to intracellular signaling. Increased matrix rigidity leads to Rac1 activation, which in turn promotes Arp2/3‐mediated actin nucleation and drives pseudopodial extension [158]. Concomitantly, integrins enriched in pseudopodia (e.g., αvβ3 and α2β1) initiate the FAK/Src signaling cascade and its downstream effectors, including MAPK/ERK and PI3K/AKT pathways, thereby facilitating tumor cell migration and enhancing cell survival [159]. Quasi‐mesenchymal PDAC, a subtype characterized by a high metastatic proclivity, exhibits exaggerated migratory and invasive potential: tumor cell clusters migrate vectorially along stiffness trajectories, breach basement membranes and form metastatic lesions in the liver [160]. Studies have also utilized the rigidity differences between tumor and normal tissues to further identify the distribution of cancerous tissue. In in vivo experiments, advanced multifrequency magnetic resonance elastography (MRE) can detect the rheological fingerprint of tumor tissue, including parameters such as elastic modulus and viscous modulus, to accurately distinguish tumors from surrounding normal host tissues [161].

At the single‐cell level, mechanical phenotypes are highly plastic. Traction force microscopy revealed that GBM cells can generate traction stress of 400 Pa on 2D glass, but only approximately 80 Pa within 3D collagen scaffolds. Remarkably, when these “3D‐acclimated” cells are placed back on a rigid substrate, they can recover a highly contractile phenotype within 6 h, indicating that cancer cells retain “mechanical memory” [162, 163]. A prognostic evaluation roadmap constructed based on these mechanical parameters combined with tumor stiffness distribution, fluidity heterogeneity, and texture features can effectively quantify tumor invasiveness and distant metastasis potential [164].

The TME can influence such potent cancer cell behaviors through multiple mechanical mechanisms. Primarily, matrix components in the TME can affect the rigidity of the entire tumor tissue. Matrix components, particularly collagen and CAFs, cooperatively determine ECM stiffness, thereby shaping the mechanical properties of tumor tissues. As the most abundant structural protein in the TME, collagen remodeling not only provides a physical scaffold for tumor growth but also activates mechanotransductive signaling pathways that govern cancer cell proliferation and invasion, ultimately driving tumor progression. In response to increased ECM stiffness, activation of the RhoA/ROCK pathway promotes nuclear translocation of YAP/TAZ. Once in the nucleus, YAP/TAZ interact with TEAD transcription factors to regulate gene programs associated with EMT, stemness (e.g., SOX2, OCT4), and invasive potential, thereby inducing phenotypic reprogramming [163, 165]. This mechanotransduction axis has been extensively characterized, particularly in breast cancer, where it plays a pivotal role in linking ECM stiffness to tumor aggressiveness [166]. Meanwhile, collagen degradation products (e.g., gelatin peptides) can serve as nutrient sources, regulate the storage and release of growth factors (e.g., VEGF, transforming growth factor [TGF]‐β), and act as key carriers of immunomodulatory signals, profoundly influencing TME structural homeostasis and tumor development processes [167, 168]. Beyond stiffness alone, emerging evidence highlights viscoelasticity—particularly stress relaxation dynamics—as an independent regulator of cancer cell behavior. Advanced glycation end products‐mediated collagen remodeling enhances ECM viscoelasticity via greater viscous dissipation and faster stress relaxation, with no observable impact on stiffness. HCC cells in such high‐viscoelastic matrices can upregulate the integrin‐β1–TLN1–YAP axis to promote proliferation and invasion [169]. Furthermore, the tissue fluidity of tumor tissue can more accurately assess the dynamic physical properties of tumors, a characteristic closely related not only to cancer cells themselves but also to the spatial distribution of other cells. Using MRE‐based collagen‐gel simulations and live‐cell real‐time tracking, studies show that recruitment and expansion of CAFs, together with ECM remodeling, like collagen cross‐linking and fiber alignment, increase stiffness and reduce tissue fluidity [164]. A meta‐analysis of MRE indicated that increased tumor tissue “fluidity” referring to high loss modulus, is positively correlated with metastasis risk, independent of genetic mutations [164]

These mechanical alterations directly induce phenotypic plasticity. High‐stiffness matrices enhance the mesenchymal phenotype of cancer cells by promoting E‐cadherin degradation and upregulating N‐cadherin and vimentin expression [170]. Reduced tissue fluidity provides mechanical guidance for the directional migration of cancer cells. Together, stiffness and fluidity constitute important mechanical inducers for the initiation of cancer cell plasticity. Importantly, these physical regulatory mechanisms do not exist in isolation but provide a basic microenvironment for subsequent chemical signal and biological cell regulation, thereby synergistically promoting cancer cell phenotypic switching.

Mechanical remodeling not only alters cancer cells themselves but also regulates immune phenotypes through direct or indirect mechanisms. A 3D constrained‐collagen platform recapitulating the plantar weight‐bearing mechanical microenvironment in melanoma demonstrated that 15% mechanical compression induced a threefold increase in γH2AX foci (a hallmark of DNA double‐strand breaks) and activated the PARP1–ERK5 axis to confer cisplatin resistance. The use of actin polymerization inhibitors can restore drug sensitivity [171]. Similarly, the physical remodeling of the ECM in HCC drives immune evasion and therapeutic resistance: At the invasive front of HCC, hypoxic metabolic myofibroblastic CAFs (myCAFs) secrete periostin (POSTN), inducing parallel alignment of collagen bundles to form 1–2 µm micropores that physically restrict CD8^+^ T cell infiltration. Meanwhile, POSTN‐integrin αvβ3 signaling inhibits NK cell degranulation, together constructing a “dual‐lock” immune privilege characterized by impaired T cell infiltration and NK cell dysfunction [172].

Beyond adaptive immune recognition, mechanosensitivity influences effector functions across both adaptive and innate immune compartments. T cells rely on their TCR to recognize short, epitopes in the context of major histocompatibility complex (MHC). Recent studies have shown that T cell activation depends on TCR–pMHC binding kinetics, and constructing a TCR function prediction map integrating mechanical parameters can potentially be used to judge TCR off‐target risk [173]. While these findings highlight the critical role of mechanical parameters in regulating T cell receptor function and activation, accumulating evidence also points to a similar mechanosensitive regulatory pattern in innate immune cells. The latest research indicates that whether macrophages fully phagocytose or nibble target cells depends on target cell cortical tension, suggesting that the physical properties of target cells themselves, rather than specific molecular signals, determine macrophage phagocytic behavior [174].

In summary, tumor mechanical remodeling exerts dual effects on cancer progression and therapy response by inducing chemotherapy resistance in cancer cells and suppressing antitumor immunity, while mechanical cues also regulate the function of adaptive and innate immune cells, emphasizing the clinical potential of mechanobiology‐based cancer therapies.

### 3.2 The “Tinting” Role of Cellular Metabolism in the TME

The chemical microenvironment of the TME, encompassing metabolites, secreted protein factors, acid–base balance status, and hypoxia levels, precisely regulates the metabolic plasticity and biological functions of cancer cells through complex crosstalk signaling networks, thereby driving dynamic phenotype switch.

#### 3.2.1 Tumor Metabolic Regulation

Metabolic heterogeneity is one of the core manifestations of cancer cells plasticity. Cancer cells can dynamically adapt to stressful microenvironments such as nutrient deprivation, hypoxia, and acidosis in the TME by rewiring or reprogramming metabolic pathways. This metabolic heterogeneity exhibits obvious temporal evolutionary characteristics with tumor progression, showing significant differences among primary, metastatic, and recurrent lesions, directly promoting cancer invasiveness and affecting treatment response efficiency [175]. Mechanistically, two interconnected features dominate: metabolism–epigenetic crosstalk and flexible switching among metabolic modes.

##### 3.2.1.1 Metabolism–Epigenetic Crosstalk as a Stabilizer of Plasticity

Cancer cell‐intrinsic metabolic reprogramming alters intracellular levels of key metabolites, which in turn act as substrates or cofactors for epigenetic enzymes, thereby regulating chromatin states and downstream transcriptional programs that control plasticity. A unifying mechanism underlying this crosstalk is that metabolic intermediates convert transient metabolic changes into stable epigenetic modifications, thereby sustaining malignant phenotypes.

Carbon metabolism‐derived metabolites represent the most well‐characterized regulators of this axis. For example, cellular ATP‐citrate lyase or nuclear ACSS2 convert glucose or acetate into acetyl‐CoA, which activates oncogenes such as *MYC* and *BCL‐2* through downstream metabolic reactions to enhance proliferation and antiapoptosis. ACSS2 inhibition reduces *MYC* enhancers acetylation and reverse the GBM stem cell‐like phenotype, validating this pathway as a potential therapeutic target [176, 177, 178]. Beyond acetylation, glycolysis‐derived lactate also functions as an epigenetic modulator: transported into the nucleus via *MCT1/4*, lactate mediates histone lactylation (e.g., H3K18la) that specifically activates transcription of EMT‐promoting genes (*SNAI1*, *TWIST*), enhancing cancer cell migration and invasion [179].

Metabolites from amino acid metabolism further expand this regulatory network by modulating histone methylation. α‐Ketoglutarate (α‐KG), a tricarboxylic acid cycle intermediate, activates JMJD family histone demethylases to promote demethylation and activation of tumor suppressor genes, while succinate accumulation inhibits JMJD family enzyme activity, leading to upregulated expression of oncogenes [180]. Additionally, NAD^+^ dictates a central metabolite linking energy metabolism and epigenetics: NAD^+^ boosters (e.g., nicotinamide riboside) restore SIRT6‐mediated H3K9 deacetylation and inhibit pancreatic cancer cell stemness, highlighting the potential of targeting NAD^+^ metabolism to reverse cancer cell plasticity [181].

Collectively, these findings support a model where metabolites act as “pigments” to spatiotemporally paint different chromatin landscapes, training cancer cells to maintain malignant gene programs. Importantly, this crosstalk is not limited to cancer cells: the TME also reshapes immune cell metabolism to drive immune evasion. For instance, fluctuations in S‐adenosylmethionine (SAM)—a universal methyl donor—modulate DNA methyltransferases (DNMTs) and HMTs, inducing methylation modification of immune checkpoint genes (e.g., PD‐1, CTLA‐4) in T cells, suppressing T cell activation and promoting tumor immune escape [182]. This dual regulation of cancer and immune cells underscores the broad impact of metabolism–epigenetic crosstalk on tumor progression.

##### 3.2.1.2 Flexible Switching Among Metabolic Modes

In parallel with epigenetic stabilization, cancer cells can flexibly switch between multiple metabolic modes such as glycolysis, OXPHOS, and fatty acid oxidation (FAO) to adapt to different TME. Signaling pathways such as HIF‐1α (hypoxia‐inducible factor‐1α), AMPK, and mTORC1 constitute the core regulatory network of this switching process [183]. Below, we dissect this switching process in the context of major TME stressors: hypoxia, nutrient deprivation, and acidosis.

Hypoxia is the canonical driver of glycolytic reprogramming, with HIF‐1α serving as the master regulator. In hypoxic TME, the stabilization of HIFs, particularly HIF‐1α and HIF‐2α, constitutes a central mechanism of cellular oxygen sensing [184]. On one hand, it promotes the switch of cancer cell metabolic mode to glycolysis (i.e., Warburg effect) by upregulating the expression of glucose transporter 1 (GLUT1) and lactate dehydrogenase A (LDHA), inhibiting mitochondrial oxidation. This effect enables cancer cells to preferentially supply energy through glycolysis even under aerobic conditions and produce large amounts of lactate to maintain the acidic environment of the TME [185]. On the other hand, HIF‐1α can also directly bind to the DNMT3B promoter and increase DNA methylation, thereby activating EMT‐associated transcription factors such as Snail and ZEB1 and sustaining stem‐like phenotypes, ultimately promoting EMT and invasive behavior, and maintain the stem cell phenotype [186]. Moreover, HIF‐2α contributes to the maintenance of stemness by regulating pluripotency factors, including OCT4 and NANOG [187]. This dual role makes HIF‐1α a key node connecting metabolism, epigenetics, and stemness.

ROS act as “second messengers” that integrate metabolic status with cell fate decisions, exerting dual regulatory effects on cancer cells. Moderate increases in ROS can activate ATM–NF‐κB to promote cancer cell survival, while excessive ROS levels trigger ferroptosis. Spatial metabolomics studies in HCC have revealed a mechanistic link between glycolysis, ROS, and ferroptosis resistance: in hypoxic, glycolysis‐high regions, ROS levels are 2.3‐fold higher than in oxygen‐rich regions, accompanied by increased lipid peroxidation product 4‐hydroxynonenal. This stress induces high expression of GPX4, forming ferroptosis resistance [169] and creating a chemical environment conducive to cancer cell survival. Therapeutically, combining GPX4 inhibitors with CAIX inhibitors induces explosive ROS accumulation under acid‐oxygen dual stress, specifically eliminating hypoxic clones [188]. Hypoxia also induce circular RNAs (circRNAs) expression, with circRNA‐encoded cPFKFB4 protein regulating pyruvate kinase M2 (PKM2) activity to further promote glycolytic reprogramming and pancreatic cancer metastasis [189].

Accompanying hypoxic stress, an acidic microenvironment arises primarily from enhanced glycolysis, with lactate efflux mediated by monocarboxylate transporters (MCT1/4) and proton extrusion via mechanisms such as V‐ATPase and carbonic anhydrase IX [190]. This acidification serves as an additional regulatory layer influencing tumor cell behavior [184]. Extracellular acidity is sensed by proton‐sensing G protein‐coupled receptors (e.g., GPR65, GPR68) and acid‐sensing ion channels (ASICs), triggering downstream pathways including ERK and PI3K/AKT to modulate cell survival, migration, and immune responses [190].

Beyond its metabolic role, lactate functions as a signaling metabolite: activation of the GPR81 axis regulates tumor growth, immune polarization, and metabolic crosstalk [191, 192, 193, 194]. In parallel, lactate‐induced histone lactylation reprograms transcriptional profiles, thereby promoting tumor plasticity and therapeutic resistance [177].

Nutrient deprivation and acidosis activate AMPK‐driven switching to OXPHOS and FAO. When nutrients are scarce and energy is insufficient in the TME, the intracellular AMPK signaling pathway is activated. It regulates downstream target proteins through phosphorylation, promotes OXPHOS and FAO, and enhances the adaptability of cancer cells to the nutrient‐deprived environment [195]. Intercellular metabolic interactions further augment this adaptive capacity: neurons transfer mitochondria to cancer cells vis tunneling nanotubes, and cancer cells acquiring exogenous mitochondria exhibit enhanced mitochondrial respiratory capacity, increased ATP levels, and improved resistance to oxidative stress, forming a subpopulation of cancer cells with higher metabolic activity [196]. In addition, when acid–base balance is disrupted in the TME, cancer cells can also drive the switch from glycolysis to OXPHOS in CRC cells through the AMPK signaling pathway, while enhancing their dependence on phosphoglycerate dehydrogenase (PHGDH). PHGDH‐mediated serine metabolism can provide essential biosynthetic precursors for cancer cells [188], forming an ecological environment conducive to cancer cell proliferation.

In conclusion, cancer cells maintain plasticity and adaptability to diverse TME stresses through two interconnected core mechanisms: metabolism–epigenetic crosstalk that converts metabolic changes into stable malignant phenotypes, and flexible metabolic mode switching that enables real‐time adaptation to environmental fluctuations. These regulatory networks not only create a TME niche favorable for tumor growth, invasion, and immune evasion but also increase the complexity of tumor progression and clinical treatment resistance. Collectively, these insights underscore the necessity of identifying context‐specific metabolic vulnerabilities—particularly the crosstalk nodes between metabolism and epigenetics and the key switches of metabolic mode transition—as promising targets for the development of novel, mechanism‐based cancer therapies.

### 3.3 Chemical Microenvironment Modulates Cancer Cell Plasticity

Beyond metabolites intermediates, secreted protein factors and the ionic microenvironment also modulate cancer cell plasticity, in part by driving aberrant epigenetic modifications. Tumorigenesis and progression are a multicellular, multisignal cascade, wherein cytokines have emerged as pivotal regulators of plasticity, exerting their effects through immune regulation and direct reprogramming of tumor cell states. For instance, the transcription factor *SOX17* maintains the self‐renewal capacity and differentiation plasticity of early‐stage tumor cells by activating Wnt/β‐catenin signaling and upregulating stem cell markers (e.g., CD44, Lgr5) [197, 198]. Notably, SOX17‐driven alterations in tumor cell plasticity further induce the secretion of immunosuppressive cytokine (e.g., TGF‐β, IL‐10) and upregulate PD‐L1 expression on the tumor cell surface [199], thereby establishing a mechanistic link between stemness programming and tumor immune escape.

TGF‐β exhibits striking spatiotemporal duality within the TME, acting as a tumor suppressor during early tumorigenesis but switching to tumor‐promoting functions during advanced progression. Its functional reversal is closely related to the differentiation state of tumor cells and interactions with TME components [200]. In HCC, for example, targeted inhibition of β‐catenin‐mediated activation of transcription factors (e.g., *IRF2*, *POU2F1*) can remodel the antitumor TME, facilitate CD8^+^ T cell infiltration, and enhance the efficacy of PD‐1 inhibitor‐based immunotherapy [201].

Acid–base balance regulation constitutes another critical chemical axis governing TME homeostasis and tumor behavior. The pH in the center of solid tumors can drop to as low as 6.2, and this acidic chemical environment modulate cancer cell malignant progression through multiple interconnected mechanisms. First, the acidic environment activates ASIC1a, causing Ca^2^^+^ influx, activating calpain–ROCK1‐β–catenin nuclear translocation, which in turn drives epithelial–mesenchymal transition (EMT) [188]. Second, elevated H^+^ can inhibit the acetylation of DNA repair protein RAD51, enhance genomic instability, and provide “fuel” for drug‐resistant mutations [186]. Third, the acidic environment skews macrophage polarization to immunosuppressive M2 phenotype and induces T cell exhaustion, thereby forming an immunosuppressive “acid wall” that hinders antitumor immunity [188]. Therapeutically, small‐molecule inhibitors (e.g., SLC‐0111) targeting carbonic anhydrase IX (CAIX), a key enzyme in acid–base balance regulation, can increase tumor pH to 6.8, restore perforin expression in CD8^+^ T cell, and synergize with anti‐PD‐1 antibody to inhibit colorectal metastasis in mice model [188].

In summary, the chemical factors orchestrate cancer cell plasticity through multiple mechanism including cancer cell metabolism, protein secretion networks, and ionic environments (see Table 3). Importantly, the regulatory mechanisms underlying these interactions exhibit distinct tumor type specificity, providing a rationale for the development of precision‐targeted therapeutic strategies tailored to different tumor entities.

**TABLE 3:** Metabolic regulation of cancer cell plasticity through epigenetic and signaling mechanisms.

Regulatory category	Core metabolite/pathway	Regulatory mechanism	Functional targets/effects	References
Metabolism–epigenetic crosstalk	Lactate	Induction of histone lactylation	Activation of EMT‐associated transcription factors, including SNAI1 and TWIST	[179]
	S‐adenosylmethionine (SAM)	Modulation of DNMT and HMT activity, promoting DNA and histone methylation	Suppression of T cell function and facilitation of tumor immune evasion	[182]
	α‐Ketoglutarate (α‐KG)	Activation of JMJD family histone demethylases	Reactivation of tumor suppressor gene expression	[202]
	Succinate	Inhibition of JMJD family demethylase activity	Upregulation of oncogenic transcriptional programs	[181]
	Acetyl‐CoA	Serves as a substrate for histone acetylation and activates acetyltransferases	Induction of oncogenes such as MYC and BCL2, enhancing proliferation and resistance to apoptosis	[176, 177]
Metabolic mode switching	Glycolytic pathway	HIF‐1α‐mediated upregulation of GLUT1 and LDHA	Hypoxia‐driven glycolytic shift and maintenance of an acidic tumor microenvironment	[203]
	OXPHOS/FAO	AMPK activation under energy stress	Enhanced adaptation to nutrient deprivation	[195]
	Glycolysis→OXPHOS	Acidosis‐induced activation of AMPK signaling	Increased dependence of colorectal cancer cells on PHGDH	[188]
	Glycolytic reprogramming	circRNA‐encoded cPFKFB4 regulation of PKM2 activity	Promotion of pancreatic cancer progression	[189]
Protein factors/epigenetic modifications	H3K4 methylation	Upregulation of H3K4 methyltransferases	Maintenance of stem‐like states in TNBC and enhanced chemoresistance	[204]
	β‐Catenin signaling	Inhibition of β‐catenin‐driven activation of IRF2 and POU2F1	Remodeling of the HCC TIME and improved immunotherapy responsiveness	[201]
	GALNT3 glycosyltransferase	Negative regulation of EGFR signaling	Suppression of cervical cancer proliferation and invasion	[205]

## 4 TME: The Common “Director” of Heterogeneity and Plasticity

In addition to cancer cells, tumor tissues harbor a diverse repertoire of stromal and immune cellular components, including vascular stromal cells, mesenchymal cells, and immune cells, which assemble into a spatially organized, functionally integrated architecture. These nonmalignant cells engage in bidirectional crosstalk via direct cell–cell contact and paracrine signaling, forming a core regulatory network that governs cancer cell plasticity. Unlike relatively static physical and chemical cues, this cellular interaction network is inherently dynamic and context dependent, serving as a central hub through which the TME orchestrates cancer cell state transitions and therapeutic resistance (see Table 4).

**TABLE 4:** Mesenchymal and immune cell populations shaping tumor cell plasticity.

Cell type	Subtype	Regulatory mechanism	Functional targets/effects	References
Mesenchymal cells (CAFs/pericytes)	CDCP1^+^FTL^+^ CAFs	Enhancement of glycolysis and iron metabolism	Increased resistance to ferroptosis and poor prognosis	[206]
	ECM‐remodeling CAFs	Secretion of POSTN and activation of the ITGAV/ITGB5–PI3K–AKT–β‐catenin axis	Induction of EMT in pancreatic ductal adenocarcinoma	[207]
	FMO2^+^ CAFs	Secretion of CCL19 and promotion of TLS formation	Increased infiltration of CD8^+^ T cells and M1 macrophages; enhanced response to anti‐PD‐1 therapy	[208]
	SPP1^+^ CAFs	SPP1–CD44 interaction and activation of AKT–mTOR signaling	Metabolic reprogramming and immune suppression in colorectal liver metastases	[209]
	Spatially conserved CAF subtypes	Defined spatial localization and neighboring cell interactions	Association with pan‐cancer patient survival	[210, 211]
	CD44^high^ tumor‐derived pericytes	Secretion of CCL2 and CSF‐1, promoting M2‐like TAM polarization	Reinforcement of stem‐like phenotypes in glioblastoma	[212]
Macrophages	Germinal‐center‐like M2 macrophages	High expression of CD163 and MRC1	Association with germinal‐center origin and poor prognosis in DLBCL	[213]
	VIM^high^ macrophages	IL‐1β secretion and activation of NF‐κB signaling in Tregs	Enhanced Treg‐mediated immunosuppression and HCC progression	[214]
	GAS6^+^ macrophages	Interaction with tumor cells via the GAS6–TYRO3 axis and modulation of propionate metabolism	Promotion of melanoma progression	[215]
	Terminally differentiated TAM lineages	Monocyte→intermediate TAM→terminal TAM differentiation regulated by TGF‐β and IL‐10	Spatial segregation between tumor parenchyma and stroma, controlling PDAC progression	[216]
T cells	TSTR cell	High expression of heat shock proteins, IL‐10 secretion	Inhibits the function of effector CD8+ T cells, promotes EMT, mediates resistance to immunotherapy	[217]
	Exhausted/activated‐exhausted CD8^+^ T cells, Treg	Inverse correlation between Th17 and Treg, regulated by TGF‐β	Forms a protumor microenvironment subtype in gallbladder cancer (GBC)	[218]
	Pre/terminal exhausted CD8^+^ T cells	IKAROS and ETS1 regulate the expression of downstream molecules	Directly affects the antitumor immune efficacy in the tumor microenvironment	[219]
B cells/DC	Extrafollicular pathway B cells	High PD‐L1 expression, IL‐10 secretion	Indicates poor tumor prognosis, mediates resistance to immunotherapy	[220]
	TAABs	High clonal expansion, interacts with activated CD4^+^ T cells (CD40–CD40L)	Regulates tumor immune evasion, can predict the response to immunotherapy	[221]
	CCR7+DC	Form DC aggregates, recruit T cells	Predict the therapeutic response of head and neck squamous cell carcinoma to pembrolizumab	[222]
Other lymphocytes	TANs	Decreased expression of activating receptors, increased expression of inhibitory receptors	Impaired antitumor function, indicates poor tumor prognosis	[223]
	Activated mast cells	Secrete MIF, form intercellular suppressive crosstalk	Enhances the resistance of pancreatic ductal adenocarcinoma to AG therapy	[224]
Microbiota	*Enterococcus faecalis, Streptococcus sanguinis*	Secrete short‐chain fatty acids, activate related signaling pathways	Promotes the progression of inflammation‐associated hepatocellular carcinoma (IM‐HCC)	[225]
	*Ralstonia spp*.	Regulate glycerophospholipid metabolism, modulate immune cell infiltration	Inhibits hepatocellular carcinoma proliferation and EMT process	[226]

### 4.1 Core Regulatory Role of Mesenchymal Cells

CAFs, as the most abundant type of mesenchymal cells in the TME, possess high heterogeneity and intrinsic plasticity. They can precisely regulate cancer cell plasticity by secreting various cytokines, chemokines, and remodeling the composition and structure of the ECM [227].

Similar to cancer cells, CAFs exhibit spatial territoriality and multiomics heterogeneity. Pan‐cancer multiomics studies have defined four conserved spatial niches of CAFs with distinct localization and biological roles: inflammatory CAFs (iCAFs), myCAFs, antigen‐presenting CAFs (apCAFs), and ECM‐producing CAFs (ecmCAFs). Each subtype occupies a specialized TME niche: iCAFs tend to distribute in perivascular spaces; myCAFs are adjacent to cancer cell nests; apCAFs reside within TLSs to exert antigen‐presenting functions; and ecmCAFs accumulate in high‐tension ECM regions, where they activate contractile gene modules via YAP/TAZ nuclear translocation to enhance cancer cell rigidity [210, 211]. This spatial‐functional specificity directly dictates their regulatory effects on cancer cells. For instance, in PDAC, ECM‐remodeling CAFs at the invasive front highly express POSTN, which bind to integrins ITGAV/ITGB5 on the surface of cancer cells, activating the PI3K/AKT/β‐catenin signaling pathway. This cascade drives EMT, enhances cancer cell migration and invasion, and induces gemcitabine resistance [207]. In breast cancer, TSPAN8^+^ senescence‐like myCAFs secrete IL‐6/8 to activate the MAPK11–RBBP6–SIRT6 axis and constructing an asparagine‐proline nutritional niche that sustains breast cancer stem cells survival and self‐renewal [228]. Beyond cell‐autonomous effects, CAFs also shape the immune microenvironment through cellular crosstalk: Spatial transcriptomics studies demonstrate that detox‐iCAFs colocalize with FOLR2^+^ macrophages to form an immune‐protective niche, while ECM‐myCAFs colocalize with TREM2^+^ macrophages to construct immune‐excluded microregions [229].

Notably, CAF subtypes are not static but undergo a programmed differentiation trajectory: they originate from normal‐like CAFs, sequentially transition to iCAFs and myCAFs, and ultimately differentiate into more invasive proliferative CAFs. This phenotypic evolution is coordinately regulated by AP‐1 family transcription factors and TGF‐β/IFN‐γ signaling [206], highlighting a dynamic regulatory axis that links TME inflammatory signals to CAF functional specialization. Collectively, CAF subtype diversity and state transitions represent a major contributor to cancer cell plasticity, offering opportunities for precision interventions targeting specific CAF functional programs rather than pan‐CAF populations.

The regulation of CAF subtypes is further characterized by tumor type and metastatic status specificity, which directly impacts therapeutic responses. In HCC, the FMO2^+^ CAF subtype secretes chemokines CCL19, which binds to the CCR7 receptor on T cells, and promotes the formation of TLSs. TLSs can recruit and activate CD8^+^ T cells and M1‐like macrophages, enhance antitumor immune responses, and thereby improve the efficacy of PD‐1 inhibitor immunotherapy [208]. In contrast, in a colorectal liver metastasis model, SPP1^+^ CAFs specifically interact with CD44^+^ cancer stem‐like cells to activate the AKT/mTOR pathway, drive glycolytic metabolic reprogramming in cancer cellls, inhibit CD8^+^ T cell infiltration, and forms an immunosuppressive niche that fosters metastatic outgrowth [209]. These studies indicate that the regulatory role of CAF subtypes is specific to tumor type and metastatic status, and some subtypes can affect treatment response by reshaping the immune microenvironment, providing new entry points for optimizing tumor immunotherapy strategies.

Beyond CAFs, pericytes represent another key mesenchymal cell population contributing to cancer cell plasticity. In GBM, pericytes can be divided into two subtypes: tumor‐derived (T‐PC) and normal‐derived (N‐PC). Strikingly, CD44‐highly expressed T‐PCs can recruit monocytes and promote their polarization to M2‐like TAMs by secreting cytokines such as CCL2 and CSF‐1. In turn, M2‐like TAMs secreted IL‐10 and TGF‐β to further enhance the stem cell‐like phenotype of GBM cells and promote tumor growth [212].

In summary, stromal cells are functionally partitioned in space and instruct cancer cell states through metabolic, mechanical, and cytokine signals. The pan‐cancer conservation of CAF spatial heterogeneity underscores its centrality, and pericyte participation further amplifies the complexity of mesenchymal regulatory networks. Immune cells, as direct effectors of tumor immunity and clinical therapy, constitute the next critical layer for understanding the bidirectional regulation of cancer cell plasticity by the TME.

### 4.2 Immune Cells: Functional Transition From “Surveillors” to “Coaches”

For decades, the TIME has been primarily regarded as a “surveillance system” dedicated to the recognition and elimination of cancer cells, with its core function oversimplified into a binary opposition between immune activation and immune suppression. However, advances in single‐cell and spatial omics technologies are systematically revising this paradigm. Mounting evidence indicates that immune cells do not merely passively “allow” tumor progression upon failure of elimination; instead, they actively participate in shaping the initiation, maintenance, and evolution of cancer cell plasticity through sustained signal input, metabolic coupling, and spatial organization. Within this framework, the immune system is increasingly recognized as transitioning from a passive “surveillor” to an active “coach” function that dynamically modulates cancer cell states during tumor evolution.

#### 4.2.1 Tumor‐Associated Macrophages

As a central immune cell population within the TME, TAMs exhibit extensive phenotypic heterogeneity. The pivotal role of TAMs is not limited to the simple promotion or suppression of tumor growth; rather, they sustainably determine the upper limit of cancer cell state plasticity via two highly coupled regulatory axes: metabolism and epigenetics [230]. This reframes macrophage polarization from a binary descriptive axis (M1–M2) into a mechanistic continuum that encodes niche‐specific “instructional” signals. Notably, immune activation is not invariably antagonistic to plasticity: inflammatory cues released by classically activated macrophages can enhance NF‐κB signaling in cancer cells and induce stemness‐associated programs (e.g., *NANOG*, *SOX2*, and *CD44*), thereby providing a priming environment for state transitions rather than enforcing terminal elimination [231]. This finding suggests that immune activation does not necessarily inhibit cancer cell plasticity; on the contrary, it may provide “priming signals” under specific contexts.

At the metabolic level, TAMs phenotypic identity is reciprocally coupled to tumor metabolic programs. Single‐cell analyses indicate that specific metabolic configurations within the TIME align with invasive behavior and therapy refractoriness, consistent with a metabolism–transcription crosstalk that stabilizes maladaptive tumor states [232]. In line with this, TAMs are better described as a continuous and interconvertible spectrum shaped by gradients such as hypoxia, nutrient availability, and matrix stiffness, rather than discrete M1/M2 bins [233]. Spatial context further refines this spectrum into functionally distinct macrophage niches. In diffuse large B‐cell lymphoma (DLBCL), macrophages occupying different tissue compartments exhibit divergent transcriptional programs; macrophages enriched in germinal‐center‐like regions show elevated expression of M2‐associated markers, such as CD163 and MRC1, correlating with cell‐of‐origin subtype and inferior overall survival [213].

Beyond their intrinsic roles, macrophages collaborate with other cell types to form specialized niches that modulate tumorigenesis and progression. The crosstalk between macrophages and cancer cells constitutes a complex regulatory network within the TME. Cancer cells can modulate macrophage polarization by secreting cytokines and other signaling molecules. In turn, macrophage‐derived exosomes act as a double‐edged sword in cancer progression, as they can both promote tumor chemoresistance and serve as potential therapeutic delivery vehicles [234]. Notably, macrophages do not merely interact directly with cancer cells; they also shape the immunosuppressive landscape by engaging with other immune subsets, thereby expanding the complexity of TME regulatory networks. For instance, in HCC, a VIM‐high macrophage subset colocalizes spatially with regulatory T cells (Tregs). IL‐1β secreted by these macrophages binds to the IL‐1R1 receptor on the Treg surface, activating the downstream NF‐κB signaling pathway. This process not only enhances the immunosuppressive activity of Tregs by upregulating PD‐1 and CTLA‐4 expression but also inhibits the cytotoxic function of effector CD8^+^ T cells, thereby creating an immune‐privileged niche that facilitating tumor progression [214]. Even more strikingly, TAM–tumor crosstalk can operate through bidirectional metabolic symbiosis. In melanoma, a more sophisticated metabolic symbiosis loop has been characterized: TEAD3 high tumor cells secrete GAS6 to activate TYRO3 on macrophages, inducing PI3K/AKT‐dependent propionate rewiring. Propionate‐containing vesicles are then transferred back to tumor cells, where H3K18 propionylation activates SOX9‐driven neural‐crest‐like plasticity [215]. This bidirectional metabolic crosstalk highlights that TAMs are not passive responders to TIME cues but active information relays that program cancer cell states.

Collectively, this bidirectional TAM–tumor cell crosstalk, combined with the time‐dependent nature of monocyte‐to‐TAM differentiation, provides a mechanistic rationale for therapeutic strategies aimed at intercepting TAM state transitions and their downstream plasticity‐inducing outputs.

#### 4.2.2 T Cells

The heterogeneity of T cell subsets represents a key regulatory node in cancer cell plasticity, with distinct T cell subsets modulating cancer cell phenotypes via diverse mechanisms. Single cell studies have categorized T cells into major subsets, including exhausted CD8^+^ T cells, effector CD8^+^ T cells, Tregs, helper T cells, and so on. Among these, exhausted CD8^+^ T cells and Tregs primarily exert immunosuppressive effects, promoting cancer cell immune evasion and plasticity transition; in contrast, effector CD8^+^ T cells secrete cytokines such as IFN‐γ and TNF‐α to inhibit cancer cell proliferation and induce apoptosis, thereby mediating antitumor functions [217]. Meanwhile, activated CD4^+^ T cells can induce inflammatory cell death in tumor cells, breaking the immune evasion barrier established by cancer cell plasticity and enabling effective control of refractory tumors [235].

Importantly, the functional states of CD8^+^ T lymphocytes in tumor exhibit a continuous differentiation spectrum: ranging from precursor exhausted states (TCF‐1^+^PD‐1^int^) with self‐renewal capacity, to intermediate exhausted states retaining partial effector functions, and ultimately to terminal exhausted states (TOX^high^TIM‐3^high^PD‐1^high^) that lose proliferative potential [236, 237]. This framework implies that the sustained engagement with tumor and cytotoxic pressure can concurrently drive progressive functional impairment of T cells, partly through the persistent induction of transcriptional regulators such as *NR4A* family members and *TOX* [236].

Beyond conventional classification, pan‐cancer T cell atlas studies have identified a distinct T cell stress response (TSTR) state, characterized by marked activation of heat shock proteins and stress signaling pathways. This state is particularly prominent following immune checkpoint blockade therapy and is highly enriched in nonresponsive patients [217]. Notably, these T cells may retain cytokine secretion capacity while failing to execute efficient cytolysis, thereby exerting sustained yet nonlethal immune pressure via mediators such as IFN‐γ and TNF‐α. Such “sublethal” pressure is increasingly viewed as a plausible driver of EMT, stemness maintenance and immune evasion programs in cancer cells. TSTR cells can secrete IL‐10 to paracrinely inhibit mTORC2 activation in adjacent effector CD8^+^ T cells, while directly upregulating ZEB1 protein translation in cancer cells via a non‐JAK/STAT pathway, thereby synchronously promoting immune evasion and the acquisition of EMT plasticity. This discovery challenges the traditional binary paradigm of “exhaustion vs. effector function,” highlighting metabolic stress memory as a novel dimension driving therapeutic resistance.

These observations underscore that T cell exhaustion is not a random or irreversible state but is precisely driven by a highly conserved network of transcriptional regulators and metabolic pathways. In the TIME dominated by effector CD8^+^ T cells, cancer cells first undergo clonal selection driven by immunoediting. T cell‐derived cytokines such as IFN‐γ and TNF‐α can induce cancer cells to upregulate antigen processing and presentation‐related molecules (e.g., TAP1, B2M, and MHC‐I) via the JAK–STAT1 pathway, increasing their susceptibility to recognition and elimination. However, upon sustained exposure to this signaling network, cancer cells can achieve immune evasion by losing MHC‐I expression, suppressing β2‐microglobulin production, or reprogramming antigen processing pathways [238]. Notably, this process is not merely “loss‐of‐function resistance” but is accompanied by cellular state rearrangement, driving cancer cells toward a phenotype with low immunogenicity and high plasticity.

Mechanistic studies further reveal that immune pressure exerted by T cells can directly induce cancer cell phenotypic plasticity transition. While IFN‐γ signaling exerts acute antitumor effects, its chronic activation can drive EMT‐related transcriptional programs via the STAT1–IRF1–ZEB1 axis, reducing cellular sensitivity to immune killing and enhancing stemness and migratory potential [217]. Likewise, combined TNF‐α and IFN‐γ signaling can engage NF‐κB and noncanonical death pathways (e.g., necroptosis, ferroptosis) that, in certain contexts, paradoxically select for cytokine‐tolerant, immune‐adapted clones [238]. As CD8^+^ T cells progress into TOX^+^ NR4A^+^ terminal exhaustion, their cytotoxic capacity declines, while their secretory output can shift toward low‐level, sustained inflammatory and stress‐associated cues. This “low‐intensity, long‐duration” exposure is increasingly recognized as a microenvironmental cue that favors reversible intermediate cancer states, characterized by immune tolerance and enhanced stemness, rather than complete tumor elimination [219].

Beyond T cell intrinsic functional states, their spatial distribution patterns further amplify the regulatory effect on cancer cell plasticity. Spatial immune architecture further amplifies the impact of T cell–cancer cell crosstalk on plasticity. Regions within TLSs are often enriched with effector T cells and APCs, providing a persistent source of immune stimulation for cancer cells. However, when TLSs are enriched with Tregs within or around their structures, they can transform from “immune amplifiers” into “immune buffers.” Tregs suppress effector T cell function via CTLA‐4, IL‐10, and TGF‐β signaling, while indirectly relieving cancer cells from lethal immune pressure, enabling cancer cells to undergo phenotypic reprogramming under sublethal immune stimulation rather than being eliminated [222, 239]. More importantly, cancer cells are not passive responders to T cell signals but actively reshape this interactive relationship. By downregulating MHC‐I and upregulating alternative death or survival pathways sensitive to cytokines (e.g., autophagy, TNF‐related signaling), cancer cells achieve “state‐switching survival” under immune pressure [238]. This mechanism reveals a core paradox of T cell–cancer cell crosstalk: strong immune pressure does not necessarily lead to tumor elimination; instead, it can serve as a critical driver of cancer cell plasticity and the evolution of immune resistance.

Taken together, T cells in tumors are not merely executors of immune surveillance. Through continuous bidirectional crosstalk, encompassing selective pressure, chronic signaling inputs and spatially organized suppression, they participate in shaping the evolving landscape of tumor cell states. This conceptual shift, from T cells as “killers” to T cells as “state sculptors,” provides a mechanistic lens for understanding immune therapy resistance, recurrence, and plasticity‐driven tumor evolution, and supports therapeutic strategies aimed at rewiring the pattern and intensity of T cell–tumor interactions rather than simply augmenting immune activation.

#### 4.2.3 B Cells and Dendritic Cells

As canonical APC population, B cells and dendritic cells (DC) play indispensable roles in adaptive immunity and represent critical components for understanding the TIME.

Compared with T cells and TAMs, the role of tumor‐infiltrating B cells in tumor biology has long been underestimated. Emerging evidence indicates that B cell states in the TIME can be categorized based on their differentiation trajectories and functional phenotypes, with distinct subsets exerting divergent effects on cancer cell plasticity. Pan‐cancer single‐cell studies have increasingly revealed that B cells within the TME do not merely mediate immunity via antibody secretion. Instead, via diverse differentiation pathways and immunomodulatory functions, they indirectly shape the landscape of cancer cell plasticity [221, 240]. Across several solid tumors, B cell states have been organized into differentiation routes that resemble extrafollicular responses versus germinal center (GC)‐like programs. Notably, extrafollicular‐associated populations, including CD11c^+^T‐bet^+^ B cells, are enriched in tumor contexts linked to immune‐checkpoint resistance and adverse outcomes [220]. These B cells indirectly impair the cytotoxicity of CD8^+^ T cells against cancer cells via high expression of PD‐L1, IL‐10, and TGF‐β, while creating a microenvironment characterized by low immune pressure but sustained inflammatory stimulation. Rather than eliminating cancer cells, this environment facilitates phenotypic reprogramming of cancer cells under sublethal immune stimulation, enhancing stemness and immune tolerance.

More critically, specific B cell subsets can directly participate in the regulation of cancer cell fate. Tumor‐associated atypical B cells (TAABs) exhibit robust clonal expansion and antibody‐secreting capacity. Autoantibodies produced by TAABs bind to surface antigens on cancer cells, forming immune complexes that activate TAMs or suppressive DCs via Fc receptor signaling, thereby indirectly altering the signaling microenvironment of cancer cells [221]. Additionally, the CD40–CD40L axis among TAABs, cancer cells, and CD4^+^ T cells can enhance local inflammatory signaling, promoting the maintenance of EMT and immune evasion‐related transcriptional programs in cancer cells. The spatial distribution of B cells also determines their regulatory direction on cancer cells. GC‐like B cells located in the core region of mature TLSs are typically associated with robust antitumor immunity; in contrast, extrafollicular B cells distributed at the TLS periphery or within the tumor bed are more prone to forming immuno‐suppressive TIME, reducing the efficiency of cancer cell elimination and providing an “immune buffer” for cancer cell state transition [239]. Thus, B cells do not simply promote or inhibit tumor progression; instead, through their subset composition, spatial localization, and secretory profiles, they exert precise and sustained regulation on cancer cell plasticity—highlighting the potential of targeting specific B cell subsets or their spatial distribution to reverse immune tolerance and enhance immunotherapy efficacy.

DC subsets modulate cancer cell plasticity by regulating antigen presentation processes and costimulatory signaling. Notably, DC are heterogeneous and can be categorized into conventional DCs (cDC1, cDC2) and plasmacytoid DCs (pDC), with distinct subsets differing in cross‐presentation capacity, costimulatory molecular expression and cytokine secretion profiles‐all of which are critical for their regulation of cancer cell plasticity. Reprogrammed cDC2 states can support effective activation of CD4^+^ and CD8^+^ T cells, increasing immune pressure and constraining diversification of tumor states. Beyond cDC2, pDC also exert antitumor effects: pDC‐derived Type I interferon programs have been reported to restrain EMT and tumor proliferation in certain contexts, highlighting that DC‐mediated signaling can directly intersect with tumor state control [241]. In contrast to these antitumor DC subsets, LAMP3^+^ DC or tolerogenic DC subsets can construct immunosuppressive TIME via molecules such as PD‐L1 and IDO, enabling cancer cells to maintain a high degree of plasticity under conditions of low cytotoxicity but sustained inflammatory stimulation. Collectively, DCs are not merely antigen‐presenting vehicles; instead, by shaping immune contexts and regulating the intensity and timing of immune pressure, thereby indirectly determine whether cancer cells are eliminated, restricted, or “tolerized” into a highly plastic, drug‐resistant state.

As pivotal cellular components of adaptive immunity, B cells and DC act as signaling hubs within the TIME. Their multilayered regulation of cancer cell plasticity not only modulates tumorigenesis and progression but also provides potential targets for tumor immunotherapy.

#### 4.2.4 Synergistic Effects of Other Immune Cells

Beyond the aforementioned key immune cells, NK cells, mast cells, and neutrophils also contribute to the formation of a multidimensional TIME, with their functional interplay weaving an intricate regulatory network that modulates cancer cell plasticity and tumor evolutionary trajectories.

Compared with peripheral blood NK cells, tumor‐associated NK cells (TANKs) exhibit significantly reduced expression of activating receptors (e.g., NKG2D, NKp30) and elevated expression of inhibitory receptors (e.g., PD‐1, TIGIT), resulting in impaired antitumor functions (e.g., cytotoxicity, cytokine secretion capacity) and strong correlations with poor patient prognosis and immune therapy resistance [223]. In the peripheral blood of healthy individuals, NK cells are divided into three major subsets (NK1, NK2, NK3) and six distinct subtypes, each with unique molecular characteristics and developmental origins. Different subtypes exhibit varying capacities for differentiation into TANKs, with the NK3 subset being most prone to conversion into functionally impaired TANKs [242]. Thus, rather than acting as unidirectional “killing tools,” the impaired cytotoxicity of TANKs leads to incomplete elimination of cancer cells and exerts sustained immune selection pressure—a key driver of cancer cell plasticity and intratumoral heterogeneity, which may still shape tumor evolutionary trajectories by favoring immune‐adapted, plastic states.

Notably, as an early warning system, mast cells also participate in the regulation of cancer cell plasticity by the TME. In PDAC, gemcitabine plus nab‐paclitaxel (AG) therapy can activate mast cells. Activated mast cells secrete macrophage migration inhibitory factor (MIF), which binds to the CD74 receptor on iCAFs, promoting iCAF secretion of cytokines such as IL‐6 and TGF‐β. Simultaneously, MIF directly inhibits the activation of CD8^+^ T cells, forming an immunosuppressive crosstalk network involving iCAFs, mast cells, and T cells, which enhances cancer cell therapeutic resistance. Targeting mast cell activation or the MIF signaling pathway can synergistically improve the efficacy of AG therapy [224]. In this context, mast cells do not merely accompany immune escape; they help convert treatment‐induced inflammation into a “buffered” niche that sustains tumor viability while facilitating adaptive state stabilization—a process that directly drives cancer cell plasticity by promoting phenotypic reprogramming and further amplifies intratumoral heterogeneity by selectively enriching therapy‐tolerant, immune‐evasive cancer cell subpopulations.

Neutrophils also exhibit significant functional differentiation and spatial dependence: in hypoxic or inflammation‐driven regions, specific tumor‐associated neutrophil (TAN) subtypes can enhance immunosuppression, promote EMT and stemness programs via IL‐1β/TGF‐β axes, driving invasive metastasis and impairing immune therapy sensitivity [243, 244]. Eosinophils, by contrast, often operate through regulation of chemotaxis, vascular permeability and local inflammatory tone, with effects that are highly context dependent: they may contribute to immune activation in some settings, but under chronic inflammation could also participate in maintaining nonlethal immune pressure that sustains phenotypic switching.

In summary, various immune cell types within the TIME do not function in isolation; instead, through multilevel nested networks of metabolic relay, cellular interaction, and antagonism, they collectively weave a dynamically evolving regulatory network. The node density and spatial configuration of this network directly determine the topological characteristics of the cancer cell plasticity landscape. Understanding the wiring logic of this network is not only the key to decoding tumor heterogeneity but also provides a precise target map for developing a “microenvironment remodeling‐first” therapeutic paradigm.

### 4.3 Regulatory Roles of Other Biological Components

In recent years, the regulatory roles of intratumoral microbiota and neural cells have gradually been recognized [245]. These components extend the regulatory landscape of the TME beyond immune and stromal compartments, introducing cross‐kingdom and neurobiological dimensions to tumor evolution.

Intratumoral microbiota exhibit pronounced spatial and functional heterogeneity and can actively shape both immune states and cancer cell phenotypes. In multifocal HCC, bacterial communities exhibit significant differences between intrahepatic metastatic‐HCC nodules, multicentric occurrence lesions, and primary HCC nodules. Among these, *Enterococcus faecalis* and *Streptococcus cardiovasculis* are enriched in IM‐HCC nodules. These two bacterial species can induce Treg infiltration and M2‐like macrophage polarization via the secretion of metabolites such as short‐chain fatty acids, constructing an immunosuppressive microenvironment. Simultaneously, they activate the TGF‐β/Smad signaling pathway to promote cancer cell EMT transition and accelerate tumor progression [225]. In contrast, the *Ralstonia* genus in HCC can alter the composition of lipid signaling molecules within the TME by regulating glycerophospholipid metabolism, while promoting M1‐like macrophage infiltration and IFN‐γ secretion to inhibit cancer cell proliferation and EMT, exerting potential antitumor effects [226]. These observations support a bidirectional model in which intratumoral microbiota act as microenvironmental engineers, coordinating metabolic chemistry with immune state to modulate cancer cell plasticity—notably, the heterogeneous distribution of pro‐ and antitumor bacteria across distinct tumor lesions further drives the spatial heterogeneity of cancer cell phenotypes.

Beyond microbial regulation, accumulating evidence highlights extensive bidirectional signal transmission exists between the nervous system and cancer cells. Cancer cells can secrete nerve growth factor to promote the neogenesis and infiltration of peripheral nerves; in turn, infiltrating nerves can release neurotransmitters (e.g., norepinephrine, acetylcholine) that activate corresponding receptors on the tumor cell surface (e.g., β‐adrenergic receptors), regulating key biological characteristics of cancer cells such as proliferation, apoptosis, and immune evasion. Pharmacological studies have suggested that β‐adrenergic blockade, as well as agents targeting dopaminergic pathways, can attenuate plasticity‐associated state switching by disrupting nerve‐TME crosstalk, with measurable antitumor effects in relevant models [246]. Bidirectional neural–tumor signal transmission adds a novel dimension to the regulation of cancer cell plasticity, and drugs targeting this interactive pathway provide new directions for tumor therapy, enriching the arsenal of comprehensive tumor treatment strategies.

**FIGURE 4:**
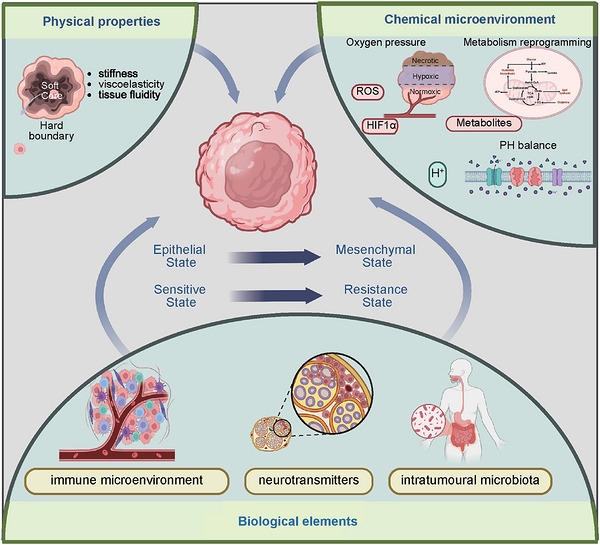
Multidimensional tumor microenvironmental regulation of phenotypic plasticity and resistance. Tumor cell behavior is profoundly influenced by diverse microenvironmental components that collectively promote plasticity and therapeutic adaptation. Physical properties of the tumor matrix, including stiffness, viscoelasticity, and tissue fluidity, impose biomechanical constraints that regulate invasion and cellular state transitions. Chemical microenvironmental factors such as oxygen deprivation, reactive oxygen species accumulation, metabolite availability, and extracellular pH imbalance drive metabolic reprogramming and stress–response signaling. Immune microenvironmental interactions further modulate tumor immunogenicity, shaping transitions between immune‐sensitive and immune‐evasive phenotypes. In addition, emerging evidence highlights the contribution of intratumoral microbiota and neuroactive signaling molecules, including neurotransmitters, in remodeling tumor progression pathways. Together, these interconnected physical, chemical, immune, and biological elements orchestrate epithelial–mesenchymal plasticity, resistance acquisition, and immune escape, forming a complex regulatory network within the tumor ecosystem.

Overall, intratumoral microbiota act as “engineers” within the microenvironment, influencing the chemical milieu of the TME via their own metabolism and the construction of microbial homeostasis. In parallel, the regulation of tumors by the nervous system extends our understanding of TME regulation beyond immune and stromal components, adding a new dimension to the modulation of cancer cell plasticity. Viewed collectively, the TME functions analogous to a “coach” in tumorigenesis and progression, exerting precise regulation on cancer cells. Only by targeting distinct regulatory nodes and transforming this multidimensional “training” into a cage that restricts tumor progression can effective tumor cure be achieved (see Figure 4).

## 5 Challenges and Insights of Tumor Heterogeneity for Precision Therapy

The spatiotemporal heterogeneity of tumors and the multidimensional regulation of the TME not only underpin the biological characteristics of tumor initiation and progression but also further clarify the molecular mechanisms driving therapeutic resistance [247]. This section systematically dissects on the fundamental reasons for the frequent failure of the “single lock‐and‐key” strategy in precision medicine and elucidates the core targeting dilemmas of precision therapy, from the dual core dimensions of “heterogeneous clones and phenotypic plasticity,” in combination with spatial and temporal heterogeneity. By reframing therapeutic resistance as a dynamic, spatio‐temporally coordinated process, we delineate the central targeting dilemmas of current precision oncology and lay the conceptual groundwork for next‐generation adaptive therapeutic strategies.

### 5.1 The Root Causes of Therapeutic Resistance

A mechanistically grounded view of drug resistance must begin with the recognition that resistance is intrinsically multidimensional and typically driven by synergistic, rather than singular, factors. In most tumors, resistance reflects the interplay between the spatial distribution of pre‐existing resistant clones, treatment‐induced phenotypic remodeling unfolding over time, and plasticity‐enabled state transitions that couple these processes [248]. Together, these forces constitute a three‐dimensional resistance system spanning an innate substrate, an acquired driver, and spatiotemporal evolution.

Within this three‐dimensional drug resistance system, pre‐existing drug resistance serves as the innate prerequisite for treatment failure, primarily driven by spatial heterogeneity. Tumors already harbor a small number of drug‐resistant clones at the time of initial diagnosis, and the spatial heterogeneity of these clones constitutes the innate basis for the development of drug resistance—unique drug‐resistant mutations can be present in the primary tumor, different metastatic lesions, and even different regions within the same lesion, forming scattered “drug‐resistant foci” [8]. Therapeutic intervention does not “induce” drug resistance but enriches these pre‐existing drug‐resistant subpopulations through selective pressure: after sensitive clones are eliminated, drug‐resistant clones that were originally present at an extremely low proportion proliferate and become dominant clones, ultimately leading to treatment failure. From a temporal perspective, such drug‐resistant clones form early in tumorigenesis and complete spatial diffusion and colonization as the tumor progresses, constituting a “temporally prepositioned reserve” of drug resistance. The “innate existence + spatial dispersion” characteristics of pre‐existing drug resistance pose challenges to precision therapy from the initial stage, while acquired drug resistance is a dynamically evolving outcome during treatment.

In contrast to the innate character of pre‐existing resistance, acquired resistance, as a dynamically evolving component of the three‐dimensional resistance system, more prominently reflects dynamic adaptation under therapeutic pressure, and its core lies in the synergy between temporal heterogeneity and plasticity. Acting as a sustained selective signal, treatment can drive epigenetic reprogramming over time, generating new resistant subpopulations without requiring new genetic mutations, through plasticity‐mediated phenotypic switching [249]. A typical example is in the treatment of EGFR‐TKIs, where cancer cells can establish a reversible drug‐tolerant state within weeks through KDM5A‐mediated histone demethylation in temporal dimension [182]. From a spatial perspective, the kinetics of this remodeling can differ across tumor regions: some niches complete epigenetic adaptation earlier and seed resistant outgrowth, which subsequently propagates through spatial expansion. This coupling of time‐dependent adaptation with spatially heterogeneous responsiveness enables resistant states to gain dominance rapidly and increases the complexity of therapeutic control. Drug‐tolerant persister (DTP) cells represent a particularly challenging manifestation of this process.

DTP cells are an extreme manifestation of cancer cell plasticity and a key hidden danger of tumor recurrence, with their drug‐resistant characteristics fully integrating spatiotemporal heterogeneity. Temporally, DTP cells can enter a low‐proliferative, reversible dormant‐like state that persists during treatment, thereby evading drug‐mediated killing; upon drug withdrawal, they can reawaken and evolve into diverse stable resistance mechanisms, although initial survival can be <0.5%, stable resistant populations can emerge after prolonged culture [250]. Spatially, DTP cells are mostly distributed in specialized niches of the TME (e.g., hypoxic regions, perivascular areas), where microenvironmental signals further enhance their dormant state and plasticity, forming scattered “recurrence seed banks.” This spatiotemporally concealed reservoir is difficult to eradicate with conventional regimens and therefore represents a central barrier to durable responses.

Importantly, this three‐dimensional resistance system is further shaped by the TME, where intratumoral heterogeneity and intercellular communication critically influence therapeutic outcomes. Tumor heterogeneity not only drives resistance intrinsically but also organizes spatial immune responses, thereby constraining immunotherapy efficacy [251]. Consistently, heterogeneity‐associated biomarkers and immune profiling strategies—including interferon‐stimulated neutrophils and anti‐PD‐1 response landscapes—have demonstrated predictive value for clinical outcomes [252, 253]. Notably, the distinct spatial and expression heterogeneity of DKK1 and CALML5 has been identified as a novel biomarker for predicting prognosis and immunotherapy sensitivity in HNSCC [254]. In parallel, TME‐resident populations, such as myeloid‐mediated networks and specific CAF subpopulations, not only establish distinct immunological niches but also express novel checkpoints (e.g., ADAM12), thereby impairing antitumor immunity [255, 256, 257, 258]. Beyond intrinsic heterogeneity, exosome‐mediated TME communication critically reinforces therapeutic resistance. Exosomal signaling, often driven by stromal components such as CAFs, promotes chemoresistance through mechanisms including miRNA transfer and ferroptosis suppression [259, 260, 261].Collectively, these observations underscore how the interplay between tumor‐intrinsic heterogeneity and TME‐mediated communication orchestrates therapeutic failure.

Ultimately, these findings indicate that therapeutic resistance emerges from the coordinated interplay of intratumoral heterogeneity, cellular plasticity, and TME‐mediated regulation. The integration of these processes—further reinforced by microenvironmental cues—renders single‐target strategies inherently insufficient. Overcoming this challenge requires a comprehensive targeting of the integrated resistance network, achieved by integrating the monitoring of clonal evolution and phenotypic drift with dynamic combinatorial interventions. Such approaches aim to simultaneously restrict pre‐existing resistant niches and limit plasticity‐driven transitions, thereby improving the precision and durability of therapeutic responses.

### 5.2 The “Targeting Dilemma” in Precision Medicine

Building on the above resistance framework, the central targeting dilemmas of precision oncology can be organized into three recurrent modes that are amplified by spatiotemporal heterogeneity and plasticity: target loss, bypass activation, and cellular identity transition [262]. These modes correspond to resistance emerging at the levels of target availability, signaling dependency, and cellular identity, respectively. Dissecting their manifestations and internal logic is a prerequisite for rational counterstrategy design.

Target loss represents the most direct dilemma in precision therapy, and its essence lies in the selective enrichment of drug‐resistant clones driven by spatiotemporal heterogeneity [263]. Targeted therapy can exert selective pressure over the temporal dimension to enrich clones with target mutations or downregulated expression; meanwhile, spatial distribution differences of such clones further exacerbate drug resistance. For example, after treatment of *EGFR*‐mutant lung adenocarcinoma (*EGFR*‐mut LUAD), cells in some regions can transdifferentiate into SCLC; following transdifferentiation, EGFR protein expression is significantly downregulated, rendering EGFR‐TKIs ineffective. Deletion or mutation of the *BCR–ABL* fusion gene can also lead to TKI treatment failure [264]. Over time, such target‐negative clones expand spatially and can overtake the lesion, explaining why durable disease control is difficult to sustain with a single target. The spatiotemporal evolutionary characteristics of target loss make it difficult for single‐target drugs to maintain long‐term efficacy.

In contrast, bypass activation constitutes a more flexible drug resistance escape mechanism for cancer cells, as it does not rely on target alteration but on plasticity‐mediated pathway switching. When the targeted pathway is blocked, cancer cells can activate alternative signaling pathways (e.g., AXL, NF‐κB, STAT3) through plasticity to sustain survival [265]. Temporally, bypass signaling can intensify with prolonged therapy exposure; spatially, different tumor regions may deploy distinct bypass programs, resulting in a heterogeneous “pathway map” [266, 267]. For example, AXL dependence in one niche and NF‐κB dependence in another. Consequently, monotherapy rarely covers the full spectrum of spatially distributed escape routes, whereas rational combinations can delay (though not always prevent) resistance.

Tumor cells can further evade lineage‐dependent targeting through cellular identity transition‐a more fundamental and irreversible escape mode that synchronizes with spatiotemporal evolution. Temporally, lineage switching typically requires weeks to months of epigenetic remodeling; spatially, it rarely occurs uniformly, instead emerging first within permissive “hotspots” such as tumor margins or regions under high microenvironmental stress, before spreading across the lesion [268]. This combination of delayed onset and spatial asymmetry makes identity transition particularly difficult to detect early and to suppress once established.

In summary, tumor spatiotemporal heterogeneity determines the “innate distribution and temporal reserve” of drug resistance, while cancer cell phenotypic plasticity provides the “acquired dynamic transition driving force” for drug resistance. The synergy between the two forms a “spatiotemporal‐plasticity” drug resistance network, rendering the “single lock‐and‐key” strategy of precision medicine ineffective in achieving durable therapeutic efficacy. To this end, it is necessary to construct a targeted next‐generation precision therapeutic strategy system to break through the treatment bottleneck.

### 5.3 New Hopes and Strategies toward Next‐Generation Precision Therapy

**FIGURE 5:**
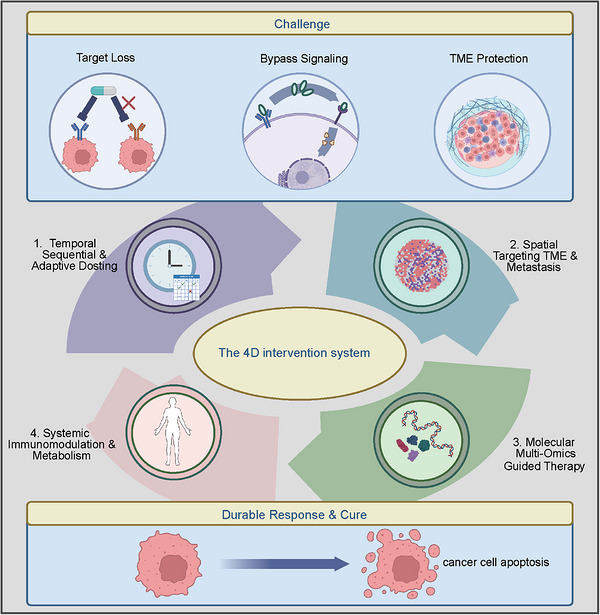
A conceptual 4D intervention framework for overcoming cancer therapeutic resistance. Cancer treatment is frequently limited by multiple resistance mechanisms, including antigen or target loss, activation of bypass signaling pathways, and protective effects mediated by the tumor microenvironment (TME). To address these challenges, a proposed 4D intervention system integrates four complementary dimensions of therapeutic control. Temporal strategies emphasize sequential, adaptive, and evolution‐informed dosing schedules to delay resistance emergence. Spatial targeting focuses on simultaneously eliminating primary tumors, metastatic sites, and supportive stromal niches. Molecular interventions employ multiomics profiling to guide precision therapy against heterogeneous tumor cell populations. Finally, systemic modulation incorporates immune reprogramming and metabolic intervention to disrupt host‐level support of tumor survival. By coordinating these temporal, spatial, molecular, and systemic strategies, this framework aims to achieve durable tumor responses and ultimately promote cancer cell elimination through apoptosis and long‐term disease control.

To address the therapeutic challenges posed by the aforementioned spatiotemporal heterogeneity and plasticity, clinical therapeutic approaches must fully account for this biological complexity. This entails refining evaluation frameworks, integrating multiomics data, and proposing a four‐dimensional strategic system of “plasticity targeting–combinatorial intervention–dynamic adaptation–technology‐driven,” which emphasizes the core supporting role of technological innovation in overcoming heterogeneity and achieving precise intervention (see Figure 5).

Innovative multiomics technologies and comprehensive databases are emerging continuously, providing technical solutions for clinical diagnosis, treatment, and prognostic assessment. Relying on single‐cell multiomics, spatial omics, artificial intelligence (AI), and other technologies, high‐resolution dynamic monitoring of tumor heterogeneity and plasticity can be achieved [269] (see Table 5). Leveraging shared database resources enables the mining of “gene‐silenced” oncogenic vulnerabilities. Clinical translational technologies such as organoids and 3D bioprinting models can be used to predict individual patient's drug resistance risk and treatment response [270], thereby guiding personalized treatment decisions.

**TABLE 5:** Methodological frameworks for dissecting tumor spatiotemporal heterogeneity and cellular plasticity.

Methodological category	Representative approach	Core application (spatial heterogeneity and plasticity relevance)	References
Multiomics integration	Vertical integration (DNA–RNA–protein/metabolite) using hierarchical network modeling and tensor decomposition	Reconstruction of causal mutation–signaling–metabolic axes, enabling coupling of driver events with adaptive responses and delineation of the temporal evolution of therapy resistance	[271]
	Horizontal integration across tumor types using spectral clustering and consensus network detection	Identification of robust pan‐cancer molecular subtypes across large multiomics cohorts, supporting cross‐organ therapeutic stratification and resolving subtype divergence driven by spatial heterogeneity	[272]
	Single‐cell multiomics integration (scRNA‐seq, scATAC‐seq and mass spectrometry imaging)	High‐resolution localization of stem‐like lineages and resistance‐associated regulatory nodes, capturing spatiotemporal heterogeneity at single‐cell resolution	[273]
Spatial omics analysis	MetaTiME framework	Integration of single‐cell transcriptomes to deconvolve tumor immune microenvironment components, identify state‐defining transcriptional regulators and resolve spatial immune heterogeneity	[274]
	MiXcan cell‐type–aware transcriptome‐wide association analysis	Prediction of cell‐type‐specific gene expression and prioritization of disease‐associated genes, mitigating confounding effects of tissue‐level spatial cellular heterogeneity	[275]
	PACpAInt deep learning model	Histopathology‐based prediction of pancreatic ductal adenocarcinoma molecular subtypes, enabling noninvasive mapping of intratumoral spatial heterogeneity	[276]
	SPIAT and spaSim spatial analysis toolkits	Quantification of spatial cell–cell patterns (colocalization, neighborhood structure), revealing prognostically relevant immune architectures within tumors	[277]
	MaCroDNA algorithm	Joint inference of single‐cell DNA and RNA sequencing data to link clonal architecture with transcriptional phenotypes and spatial clone distribution	[278]
	Starfysh computational toolbox	Integration of histological imaging with spatial transcriptomics to define tissue‐specific cellular states and identify tumor–immune spatial hubs as heterogeneity hotspots	[279]
Single‐cell sequencing and functional profiling	Droplet‐based single‐cell Hi‐C	High‐throughput profiling of chromatin conformation to track clonal dynamics and oncogenic events, resolving the temporal emergence of resistant clones	[279]
	Multimodal single‐cell platforms (nanoparticle‐assisted LDI‐MS combined with protein quantification)	Characterization of metabolic trajectories underlying CD8^+^ T cell exhaustion, capturing temporal evolution of immune functional heterogeneity	[280]
	Modular and detachable DNA assembly systems	Quantification of RNA family composition and discrimination between normal and malignant cells, highlighting spatially heterogeneous tumor‐specific expression programs	[281]
	Single‐cell Ca^2^^+^ imaging combined with graph‐based unsupervised learning and neural networks	Identification of heterogeneous oncogenic Ca^2^^+^ signaling states, prediction of chemoresistant cancer cells and mapping of their spatiotemporal evolution	[282]
Artificial intelligence and machine learning models	CeiTEA adaptive hierarchical clustering	Resolution of complex hierarchical relationships among cell types and states, outperforming conventional clustering in identifying spatiotemporally heterogeneous subpopulations	[283]
	tumor microenvironment (TME) classifiers	Stratification of tumors into immune‐excluded, immune‐suppressed, or immune‐activated categories, guiding personalized immunotherapy and addressing spatial immune heterogeneity	[284]
	MSFusion multisource feature fusion network	Integration of deep‐learning‐derived and handcrafted features to improve grading of invasive breast cancer and refine heterogeneous subtype classification	[285]
	stClinic dynamic modeling framework	Integration of multislice spatial multiomics data to reveal clinically relevant tumor ecological niches and niche‐driven spatial resistance patterns	[286]
	CellPhenoX interpretable machine learning	Identification of cell‐type‐specific phenotypes associated with clinical outcomes, linking single‐cell states to disease progression and temporal phenotypic heterogeneity	[287]
Clinical translation and model validation	Patient‐derived organoid (PDO) models	Integration of multilesion biopsies to predict drug response in prostate cancer, modeling the impact of spatial heterogeneity on therapeutic outcome	[288]
	3D‐bioprinted gastric cancer models (3DP‐GC)	Personalized prediction of chemotherapeutic efficacy and simulation of tumor spatial architecture, enabling high‐throughput drug screening	[289]
	NeoDisc proteogenomic pipeline	Integration of immunopeptidomics, genomics, and transcriptomics to prioritize tumor‐specific antigens and overcome antigen‐expression heterogeneity	[290]

With the support of these technologies, on the one hand, targeting cancer cells themselves can be achieved through the combination of multiple targeted agents, implementing a “block‐lock” strategy. For instance, combining epigenetic drugs with conventional therapies to “lock” cancer cell states and prevent phenotypic escape; developing inhibitors targeting key nodes of EMT transcription factors or stem cell pathways to directly target the core of plasticity regulation and block spatiotemporal phenotypic transitions. Combining targeted drugs with differentiation inducers (e.g., retinoic acid) can both inhibit tumor proliferation and “lock” cell states; utilizing the synergistic effect of SH2 domain‐containing inositol 5′‐phosphatase 2 and polo‐like kinase 1 inhibitors to suppress esophageal squamous cell carcinoma growth and avoid drug resistance [291]. On the other hand, targeting the TME can be accomplished by remodeling the TME using nanozymes and multifunctional nanoplatforms to enhance the efficacy of chemotherapy and immunotherapy. For example, the use of nanozyme‐immunomodulator dual‐mode nanoparticles (l‐arginine + PEG‐Pt) simultaneously alleviates hypoxia and acidosis, increasing the antiprogrammed PD‐1 response rate from 15 to 48% [292, 293]. Utilizing humanized anti‐POSTN monoclonal antibodies to break the collagen physical barrier, increase tissue fluidity, recruit TLSs, and promote antitumor immunity.

In addition, during the treatment process, based on the evolutionary rules of tumor spatiotemporal heterogeneity, “treatment holidays” and drug rotation models should be adopted: adjusting treatment regimens according to dynamic changes in tumor burden and circulating tumor markers (e.g., cell‐free DNA, circulating tumor RNA) [294, 295, 296]. With the goal of “controlling tumors rather than eradicating them,” this approach delays the spatiotemporal expansion and evolution of drug‐resistant clones.

Collectively, precision oncology is undergoing a conceptual transition—from “a single key” to “a dynamic locksmith,” by leveraging the trinity technical matrix of single‐cell–spatial–AI to incorporate heterogeneous clones and phenotypic plasticity into a closed loop of real‐time monitoring and combinatorial intervention. The future clinic will feature “digital twin tumors”—virtual models for each patient that continuously receive multiomics data streams, with AI engines instantly outputting the optimal drug combination and administration schedule, truly realizing adaptive precision medicine that “leaves tumors with no way to escape” [297].

Nevertheless, the clinical translation will likely be incremental and modular, rather than emerging overnight as a fully comprehensive virtual replica of each patient's tumor. A more realistic near‐term path may be to integrate multiomics features with patient‐specific computational models to predict clonal expansion, treatment sensitivity, and resistance emergence, with these models serving not as autonomous decision engines but as iterative decision‐support tools. In glioma, isotope tracing, stoichiometric simulations, and machine learning were integrated to develop a digital twin framework for in vivo metabolic flux estimation, illustrating the feasibility of disease‐specific, function‐focused proof‐of‐concept applications in oncology [298]. Likewise, a physics‐informed machine learning digital twin for prostate cancer monitoring reconstructed tumor growth trajectories from longitudinal PSA data, suggesting that early clinical utility may first emerge in focused monitoring settings [299]. More broadly, perspectives in mathematical oncology suggest that mechanistic and evolutionary models may serve as clinically testable decision‐support tools for adaptive scheduling, dose optimization, and personalized treatment design [300]. Together, these observations suggest that tumor digital twins are more likely to first emerge as modular frameworks integrating biomarkers, biologically informed modeling, and dynamic treatment adjustment, with adaptive therapy representing one of their earliest clinically actionable forms. However, broader implementation will require standardized integration of multiomics, imaging, and clinical data, stronger cross‐center validation of computational models, and workable frameworks for regulatory acceptance and routine workflow integration [300]. Therefore, further progress will depend not only on advances in computation, but also on standardized data infrastructures, prospective interventional studies, and clinically tractable validation strategies.

## 6 Summary and Outlook

Tumor heterogeneity was once regarded as a “noise” confounding precision therapy, but it has now evolved into a core “clue” for decoding the evolutionary rules of cancer. Over the past decade, single‐cell and spatial omics technologies have enabled us to gradually decipher a critical insight: a single tumor harbors a five‐dimensional landscape encompassing genomic, epigenomic, transcriptomic, proteomic, and metabolomic heterogeneities, which undergo real‐time remodeling under temporal selection pressures (e.g., spontaneous tumor progression, therapeutic interventions).

In this review, we systematically delineate the multidimensional core features of tumor heterogeneity, spanning intertumoral heterogeneity, and intratumoral multiomics heterogeneity in the spatial dimension, to temporally dynamic heterogeneity arising from both spontaneous evolution and therapy‐induced selection, as well as the “spatiotemporal dual divergence” between primary and metastatic lesions. By integrating these layers of heterogeneity, we comprehensively elucidate the innate distribution basis and dynamic accumulation patterns of therapy‐resistant clones. Furthermore, we clarify the pivotal “instructive” role of the TME, which drives cancer cell plasticity and phenotypic switching through a multifaceted regulatory network involving physical–mechanical remodeling, chemical–metabolic modulation, and biological cellular crosstalk.

Against this theoretical backdrop, our next imperative is to advance cancer research from the stage of “visualizing heterogeneity” to predicting and manipulating it precisely, leveraging cutting‐edge technologies for dynamic capture and monitoring of cancer progression. This shift will facilitate the paradigm upgrade of precision oncology from “static target‐oriented therapy” to “dynamic ecosystem‐based modulation.” Conventional “one‐to‐one” targeted strategies are inherently insufficient to halt cancer progression, as they fail to counteract the synergistic effects of spatiotemporal heterogeneity and cellular plasticity.

Therefore, future efforts should focus on three interconnected directions. First, accelerate the integration and translational application of multiomics technologies, and facilitate the in‐depth integration of AI with multiomics datasets to construct “digital twin tumor” models, enabling real‐time and dynamic monitoring of tumor heterogeneity and plasticity. Second, optimize clinical translational platforms such as patient‐derived organoids (PDOs) and 3D bioprinting, and combine these tools with multilesion biopsy and liquid biopsy techniques to enhance the accuracy of treatment response prediction, thereby supporting the dynamic optimization of personalized therapeutic regimens. Third, promote the clinical implementation of innovative strategies: based on the concept of “adaptive therapy,” conduct multicenter clinical trials to validate the efficacy of a four‐dimensional therapeutic strategy system (plasticity targeting, combinatorial intervention, dynamic adaptation, and technology‐driven); explore synergistic combinations of plasticity‐targeting agents with immunotherapy and antiangiogenic therapy; and harness core database resources (e.g., Cancer Dependency Map and Single‐Cell Atlas of Cancer) to facilitate the sharing and translational application of therapeutic insights across different cancer types.

Ultimately, these endeavors aim to reduce tumor recurrence rates and improve patient survival outcomes. With the advancement of these research directions, we anticipate a transformative breakthrough that will overcome the bottlenecks of traditional precision therapy, ushering in a paradigm shift and new clinical hope for cancer treatment.

## Author Contributions

Zhen Wang and Chenghui Yang conceptualized and designed the study. Hanwen Hu and Zhixing Hao drafted the manuscript. Hanwen Hu and Lili Li designed and prepared the figures. Zhixing Hao and Huiying Liu performed the literature search and data collection. All authors have read and approved the final manuscript.

## Funding

This work was supported by grants from the National Natural Science Foundation of China (82273449, 82302901), “Pioneer” and “Leading Goose” R&D Program of Zhejiang (2026C02A1111), Medical and Health Science and Technology Project of Zhejiang Province (2024KY1039, 2024KY1250), Beijing Xisike Clinical Oncology Research Foundation (Y‐Young2023‐0112), and the Special Funding Project for Characteristic Directional S‐Disciplines of The First Affiliated Hospital of Wenzhou Medical University (wyyy‐2025S01).

## Ethics Statement

The authors have nothing to report.

## Conflicts of Interest

The authors declare no conflicts of interest.

## Data Availability

The data generated in this study are available within the article and its supplementary data files.
